# Bovine meat and milk factor protein expression in tumor‐free mucosa of colorectal cancer patients coincides with macrophages and might interfere with patient survival

**DOI:** 10.1002/1878-0261.13390

**Published:** 2023-02-22

**Authors:** Ekaterina Nikitina, Amelie Burk‐Körner, Manuel Wiesenfarth, Elizabeth Alwers, Danijela Heide, Claudia Tessmer, Claudia Ernst, Damir Krunic, Petra Schrotz‐King, Jenny Chang‐Claude, Moritz von Winterfeld, Esther Herpel, Alexander Brobeil, Hermann Brenner, Mathias Heikenwalder, Michael Hoffmeister, Annette Kopp‐Schneider, Timo Bund

**Affiliations:** ^1^ Division of Episomal‐persistent DNA in Cancer‐ and Chronic Diseases German Cancer Research Center (DKFZ) Heidelberg Germany; ^2^ Division of Biostatistics German Cancer Research Center (DKFZ) Heidelberg Germany; ^3^ Division of Clinical Epidemiology and Aging Research German Cancer Research Center (DKFZ) Heidelberg Germany; ^4^ Division of Chronic Inflammation and Cancer German Cancer Research Center (DKFZ) Heidelberg Germany; ^5^ Monoclonal Antibody Unit of the Genomics and Proteomics Core Facility German Cancer Research Center (DKFZ) Heidelberg Germany; ^6^ Light Microscopy Facility German Cancer Research Center (DKFZ) Heidelberg Germany; ^7^ Division of Preventive Oncology German Cancer Research Center (DKFZ) and National Center for Tumor Diseases (NCT) Heidelberg Germany; ^8^ Unit of Genetic Epidemiology German Cancer Research Center (DKFZ) Heidelberg Germany; ^9^ Cancer Epidemiology Group, University Medical Center Hamburg‐Eppendorf University Cancer Center Hamburg Hamburg Germany; ^10^ Institute of Pathology University Hospital Heidelberg Germany; ^11^ Pathologie Rosenheim Germany; ^12^ Tissue Bank of the National Center for Tumor Diseases (NCT) Heidelberg Germany; ^13^ German Cancer Consortium German Cancer Research Center Heidelberg Germany

**Keywords:** bovine meat and milk factors, chronic inflammation, colorectal cancer, infectious indirect carcinogenesis

## Abstract

Bovine milk and meat factors (BMMFs) are plasmid‐like DNA molecules isolated from bovine milk and serum, as well as the peritumor of colorectal cancer (CRC) patients. BMMFs have been proposed as zoonotic infectious agents and drivers of indirect carcinogenesis of CRC, inducing chronic tissue inflammation, radical formation and increased levels of DNA damage. Data on expression of BMMFs in large clinical cohorts to test an association with co‐markers and clinical parameters were not previously available and were therefore assessed in this study. Tissue sections with paired tumor‐adjacent mucosa and tumor tissues of CRC patients [individual cohorts and tissue microarrays (TMAs) (*n* = 246)], low‐/high‐grade dysplasia (LGD/HGD) and mucosa of healthy donors were used for immunohistochemical quantification of the expression of BMMF replication protein (Rep) and CD68/CD163 (macrophages) by co‐immunofluorescence microscopy and immunohistochemical scoring (TMA). Rep was expressed in the tumor‐adjacent mucosa of 99% of CRC patients (TMA), was histologically associated with CD68^+^/CD163^+^ macrophages and was increased in CRC patients when compared to healthy controls. Tumor tissues showed only low stromal Rep expression. Rep was expressed in LGD and less in HGD but was strongly expressed in LGD/HGD‐adjacent tissues. Albeit not reaching statistical significance, incidence curves for CRC‐specific death were increased for higher Rep expression (TMA), with high tumor‐adjacent Rep expression being linked to the highest incidence of death. BMMF Rep expression might represent a marker and early risk factor for CRC. The correlation between Rep and CD68 expression supports a previous hypothesis that BMMF‐specific inflammatory regulations, including macrophages, are involved in the pathogenesis of CRC.

## Graphical abstract

BMMFs are a group of infectious agents related to indirect carcinogenesis. Macrophage expression of the conserved BMMF Rep protein in tumor‐adjacent mucosa of CRC patients is increased compared with expression in the tumor and mucosa of healthy donors, indicating that BMMF is involved in inflammation‐driven, indirect carcinogenesis of CRC (zur Hausen 2009). Even after resection and therapy, Rep expression persists and might favor ongoing chronic inflammation, tumor recurrence and metastasis. Rep might, therefore, serve as prognostic marker for CRC.

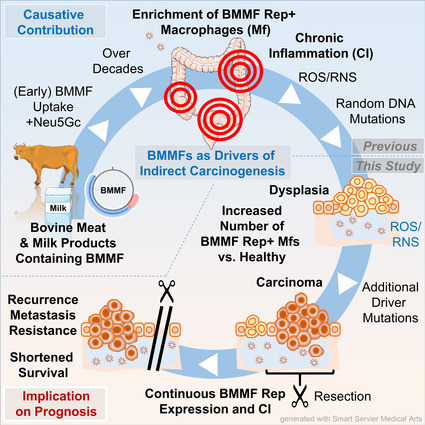

**Abbreviations**
AB: antibody
BMMF: bovine meat and milk factor
CRC: colorectal cancer
DAB: diaminobenzidine
FFPE: formalin‐fixed paraffin‐embedded
H&E staining: hematoxylin & eosin staining
HGD: high‐grade dysplasia
HR: hazard ratio
HRP: horseradish peroxidase
IHC: immunohistochemistry
INT: intensity
LGD: low‐grade dysplasia
Mf: macrophage
POS: positivity
Rep: replication protein
RNS: reactive nitrogen species
ROS: reactive oxygen species
TMA: tissue microarray

## 1 Introduction

Bovine meat and milk factors (BMMFs) are episomal, plasmid‐like DNA molecules isolated from bovine sera and milk products and from the peritumor of colorectal cancer CRC patients amongst others [1, 2, 3, 4, 5, 6, 7, 8, 9, 10]. BMMFs were proposed as zoonotic infectious agents and risk factors involved in indirect cancerogenesis of several types of cancer including CRC [11, 12, 13, 14]. Our studies specifically focus on a link between BMMF and colorectal cancer and are supported by an epidemiological association between global consumption of bovine (red) meat and dairy products and colorectal cancer incidence [11, 12, 15, 16, 17, 18]. The effect of high bovine milk consumption on CRC incidence and mortality was studied intensively throughout adult life, showing no effect or a decrease in CRC incidence or mortality, while several individual studies also show an increased CRC risk [19]. One large study specifically focusing on milk consumption during the first years of life concluded that early high milk consumption represents a strong risk factor and triples the odds for CRC [20]. A correlation of high (adult) milk consumption with increased cancer risk has already been reported for cancers like breast, lung and prostate cancer [13].

CRC is the third most common cancer in men and the second most common cancer in women [21]. CRC incidence (1.03 million for men and 817 000 for women worldwide in 2018) is predicted to increase by 72% and 82%, respectively, by 2040 [22]. CRC ranks at position 4 for male and position 3 for female patients with respect to mortality [22]. A number of lifestyle and environmental contributions such as dietary habits, smoking, excessive alcohol consumption, lack of physical activity and obesity are known to increase cancer risk [18, 23, 24, 25, 26, 27, 28, 29]. In addition, a smaller fraction of inherited colon cancer risk factors (like Lynch syndrome or familial adenomatous polyposis) contributes to CRC [30, 31]. Still, we know little about additional systemic or infectious risk factors contributing to colon cancer. In the context of inflammatory bowel diseases (IBD) and its two most frequently diagnosed variants Morbus Crohn and colitis ulcerosa, a chronic inflammatory bowel environment leads to a significantly increased risk of colon cancer [32, 33].

In the past and in this presented study, we analyzed a possible causative association of BMMF and CRC along with additional cancer types such as, e.g. lung, breast, liver and prostate cancer [11, 12, 13, 15, 17, 34]. We already outlined a hypothesis on BMMF‐specific indirect carcinogenesis for CRC based on expression of a BMMF‐encoded protein (Rep), the induction of local chronic inflammation in interstitial tissues followed by prolonged production of diffusing reactive oxygen and nitrogen species (ROS/RNS) [11, 16]. We further hypothesized (and in part already showed) that ROS/RNS increase DNA mutations in replication‐competent cells of the colonic crypts by oxidative changes (and even in BMMF DNA laser microdissected from affected tissues) exemplified by 8‐hydroxydesoxyguanosin (8OHdG) staining, a surrogate marker for oxidative DNA mutation [11, 13, 16]. These cells, which have most likely never been in contact with BMMF themselves, are expected to represent the precursors for dysplasia, polyps and development of CRC. BMMF DNA closely related to the replication‐competent and bioactive BMMF1 isolate H1MSB.1 [5, 6, 35], which was originally identified in milk, was specifically isolated from peritumoral interstitial cells between the crypts of Lieberkühn in tissues of CRC patients, but not from CRC tumor material [6, 13].

H1MSB.1 (1768 nt) comprises specific repeats (iterons) involved in DNA replication and a conserved, putative replication gene encoding for a 324 aa protein (Rep) [5, 6]. In histological stainings of CRC tissues, mouse monoclonal antibodies (AB) raised against the Rep showed Rep expression in macrophages of the mucosal, interstitial lamina propria adjacent to the tumor, but not in the tumor itself [6, 13]. Antibodies AB3 and AB10 showed highest tissue reactivity and also detected overexpressed H1MSB.1 Rep (in HEK293TT cells) dose dependently in immunoblotting and after formalin fixation based on immunohistochemistry (IHC) (Fig. S1, antigen and antibody dose dependency of AB3/AB10 detection was shown in immunofluorescence microscopy; specificity of IHC antigen reaction was tested by antigen competition on CRC tissues) [13].

Here, the same mouse monoclonal antibodies AB3 and AB10 were used to quantify Rep expression based on a clinical cohort of tissues from healthy colonic or rectal mucosa, tumor and tumor‐adjacent mucosa of CRC patients as well as dysplastic tissues to follow Rep expression over the adenoma‐to‐carcinoma sequence and to allow case‐versus‐control analyses. A tissue microarray (TMA) of CRC patients offering paired tumor and tumor‐adjacent mucosa was used to analyze an association of Rep (and CD68) expression with clinic‐epidemiological parameters including patient survival.

## 2 Material and methods

### 2.1 Clinical cohort of CRC tumor and tumor‐adjacent tissues, dysplasia and healthy individuals

Detection of BMMF Rep expression was analyzed based on formalin‐fixed, paraffin‐embedded (FFPE) and pathologist validated tissue sections from healthy (*n* = 10, no obvious disease and inflammation background), low‐ and high‐grade dysplasia (LGD and HGD, *n* = 11 and *n* = 18, respectively, including tissue adjacent to the dysplasia), tumor and paired tumor‐adjacent mucosa of CRC patients (*n* = 26, each) based on a cohort approved by the ethics committee of the Medical Faculty Heidelberg under the ethics vote S‐206/2005. Written informed consent was given by all participants. The tissues were provided by the Tissue Bank of the National Center for Tumor Diseases (NCT) Heidelberg, Germany, in accordance with the regulations. The study methodologies conformed to the standards set by the Declaration of Helsinki.

### 2.2 TMA patient cohort

Association between BMMF Rep expression and clinical parameters in colorectal cancer was analyzed based on a cohort of patients participating in the DACHS study (*Darmkrebs: Chancen der Verhütung durch Screening; Colorectal Cancers, Chances for Prevention through Screening*). DACHS is an ongoing population‐based case–control study on colorectal cancer with additional follow‐up of cases, which has been conducted in the Rhine‐Neckar region of Germany since 2003. A detailed description of the study has been published before [36, 37]. The DACHS study has been approved by the ethics committees of the University of Heidelberg and the state medical boards of Baden‐Württemberg and Rheinland‐Pfalz (Ethics vote S‐310/2001). Written informed consent was given by all participants. For the current analysis, we selected patients with a first diagnosis of CRC who were recruited at University Hospital in Heidelberg in 2003 and 2004. DACHS tissue samples were provided by the Tissue Bank of the National Center for Tumor Diseases (NCT) Heidelberg, Germany, in accordance with the regulations of the tissue bank and the approval of the ethics committee of Heidelberg University. The study methodologies conformed to the standards set by the Declaration of Helsinki. Analysis of the DACHS cohort was based on immunohistochemistry of 10 individual tissue microarrays (TMAs) with consecutive cuts for the different staining conditions (see below). The TMAs consisted of (paired) duplicates offering both tumor and the corresponding tumor‐adjacent mucosa of the same donor based on formalin‐fixed paraffin‐embedded (FFPE) tissues cuts. From the initial number of 259 patients, 13 were excluded because of insufficient tissue quality or missing tissue spots or missing clinical data. For validation of BMMF Rep antibody staining, two TMA sections of the DACHS cohort together with a set of > 20 samples of paired tumor and tumor‐adjacent mucosa tissues were used and subjected to isotype control staining.

### 2.3 Ascertainment of patient and medical factors and mortality follow‐up

Data on sociodemographic, lifestyle and dietary factors as well as medical history of patients were obtained by personal interviews using standardized questionnaires, which were conducted by trained interviewers during hospitalization, typically a few days after first CRC surgery or shortly after hospital discharge (details for the TMA cohort are described in [37]). Tumor characteristics and other medical data, including tumor site and UICC tumor stage (I–IV), recurrences and details of treatment were obtained from hospital discharge letters, pathology reports and reports of previous large bowel endoscopies as well as follow‐up questionnaires sent to patients' doctors approximately 3 years after diagnosis. Additional information was collected 5 years after diagnosis from the patients alive, including information on newly diagnosed diseases and recurrences (verified by medical records by the physicians). Information on vital status and date of death were extracted based on the population register. Causes of death were verified by death certificates (from the health authorities in the Rhine‐Neckar region) and filed according to World Health Organization guidelines. The follow‐up time was calculated as the time between the date of diagnosis and the date of event or censoring. Follow‐up time of patients with no event of interest (death and recurrence) was censored at the date of the last follow‐up.

Acquisition of dietary habits based on questionnaires was categorized for meat consumption (never, once or less than once a week, at least several times a week and at least once a day) and milk consumption (once or less than once a week, at least several times a week and at least once a day).

### 2.4 Tissue staining

FFPE sections were stained with primary anti‐BMMF1 Rep (monoclonal AB3 and AB10, DKFZ Heidelberg, Baden Wurttemberg, Germany, 1 : 1 mix), anti‐CD68 (Cell Signaling, Danvers, MA, USA, # 76437) and anti‐CD163 (Novusbio, Centennial, CO, USA, NB110‐40686) antibodies overnight at room temperature in a dilution of 1 : 250 (Rep), 1 : 500 (CD68) and 1 : 200 (CD163) as described previously [13]. Secondary goat anti‐mouse Alexa Fluor 594 (Invitrogen, Waltham, MA, USA, #A11032), goat anti‐rabbit Alexa Fluor 488 (Invitrogen, #A11034) and donkey anti‐mouse Alexa Fluor 647 (Invitrogen, #A31571) were incubated for 60 min at room temperature. For negative control staining, PBT diluent containing PBS, BSA, Tween20 and sodiumazide was used. TMAs were stained fully automatically on a BOND MAX machine (Leica Biosystems, Nussloch, Baden Wurttemberg, Germany) with EDTA epitope retrieval buffer. Primary antibodies anti‐BMMF1 Rep (monoclonal AB3, DKFZ Heidelberg) and anti‐CD68 (Cell Signaling, #76437) antibody were incubated for 30 min at room temperature in a dilution of 1 : 500 (4 μg·mL^−1^, Rep) or 1 : 1000 (CD68). Secondary rabbit anti‐mouse (Abcam, Cambridge, UK, #125904) was incubated for 20 min at room temperature. Detection was performed by using Bond Polymer Refine Detection Kit (Leica Biosystems, #DS9800) including DAB chromogen and hematoxylin counterstain. Both individual and TMA slides were scanned with a Hamamatsu Nanozoomer slide scanner (Hamamatsu, Shizuoka, Japan) and analyzed with ndp.view2 plus software (Hamamatsu).

### 2.5 Tissue analysis

Semi‐automated quantification of Rep, CD68 and CD163 staining of individual FFPE tissues was performed with an in‐house developed script for imagej/fiji (v1.52) [38] based on signal intensity of DAPI nuclear and antibody staining of interstitial cells in digitalized scans after Rep/CD68/CD163 co‐immunodetection. Cell density was measured in 3–7 randomly selected microscopic areas, in which specific cells within the lamina propria were analyzed (excluding epithelial cells and luminal parts of the crypts). Tumor core was included for analysis of tumor samples of CRC group. On average, 4300 nuclei per sample within a total area of approximately 2 mm^2^ were included for all samples, except for the size‐limited adjacent tissues of dysplasia (~ 1600 nuclei per sample). The data were represented as a % of Rep, CD68‐, CD163‐positive cells and/or its combinations normalized either to total number of nuclei or total number of macrophages (CD68‐ and/or CD163‐positive cells) and compared with previous quantifications [13] and literature (CD68/CD163) to assess objective data analysis.

For analysis of BMMF1 Rep and CD68 tissue staining on the consecutive TMA slides, the staining was semi‐quantitatively scored according to two parameters: the percentage of stained cells (POS) and intensity (INT) were determined. POS of BMMF1 Rep staining was assessed based on cut‐off levels for the fraction of stained cells which were semi‐empirically set up after review of the tissue spots (blinded for any clinical information) to reproducibly differentiate the following POS levels: 0 = no detection (truly negative), 1 = 1–10% positive cells (insecure staining of a low number of cells), 2 = 11–30% positive cells (intermediate number of stained cells) and 3 = > 30% positive cells (high number of stained cells, soring examples given in Results section). Due to the generally increased level of CD68 staining, the cut‐offs for CD68 POS1/2/3 were doubled: 0 = no staining, 1 = < 20% positive cells, 2 = 20–60% and 3 = > 60% of positive cells. Intensity (INT) for both stainings (BMMF1 Rep and CD68) was graded as follows: 0 = no detection, 1 = moderate staining and 2 = intense staining. Scoring was performed blinded for clinical parameters by two independent raters (AB‐K and TB) in < 3 days (to maintain interpretation consistency) followed by a subsequent “on screen” agreement on one INT and POS score.

### 2.6 Statistical analysis

Statistical analysis of cell‐based Rep/CD68/CD163 positivity in the individual healthy, LGD, HGD and CRC samples was performed based on Wilcoxon rank‐sum test for unpaired samples and Wilcoxon signed‐rank test for paired samples (dysplastic vs. adjacent). *P*‐values were adjusted for multiplicity by using Holm correction separately by outcome variable and groups of tests, that is adjustment within group “healthy vs. all other groups”, “all pairs of dysplastic LGD, HGD, tumor”, “all pairs of LGD‐adjacent, HGD‐adjacent, tumor‐adjacent” and group “paired samples dysplastic vs adjacent”.

Correlation of BMMF1 Rep and CD68 staining in the TMA data was determined by Spearman's rank correlation coefficient rho. Association between INT/POS and clinical data was tested using Kruskal–Wallis tests for nominal variables and Jonckheere–Terpstra tests for ordinal variables such as age group (grouped into 37–55, 56–65, 66–79 and 80–98 years), tumor stage (UICC stages I–IV), meat consumption (never: “no”; once or less than once a week: “low”; at least several times a week: “medium”; and at least once a day: “high”) and milk consumption (once or less than once a week: “low”; at least several times a week: “medium”; and at least once a day: “high”) with Holm's adjustment for multiple testings.

Cumulative incidence curves truncated at 10 years follow‐up time were used to assess distributions of CRC‐specific and non‐CRC‐specific and overall deaths. Curves were compared by using Gray (log‐rank type) tests for equality of cumulative incidence curves. To assess the effect of BMMF1 Rep and CD68 on overall survival and cause‐specific survival adjusted for possible confounders, multivariable Cox (cause‐specific) proportional hazards regression models were fitted based on 246 observations. Models included the following covariates: age (grouped into 37–55, 56–65, 66–79 and 80–98 years), gender, tumor localization (colon or rectum), tumor stage (UICC stages I–IV), therapy (patients treated with neoadjuvant‐, chemo‐ or radiotherapy or not), microsatellite instability (MSI) state (microsatellite stable (MSS) or MSI high), milk consumption (no, low, medium and high), meat consumption (low, medium and high) and BMMF1 Rep or CD68 detection (Rep and CD68 INT and POS). Analyses were performed using r‐4.1.0 [39] using packages cmprsk 2.2–10, desctools 0.99.42 and prodlim 2019.11.13.

## 3 Results

### 3.1 Rep expression in CRC adjacent mucosa is increased compared to expression in the tumor and mucosa of healthy donors

**Fig. 1:**
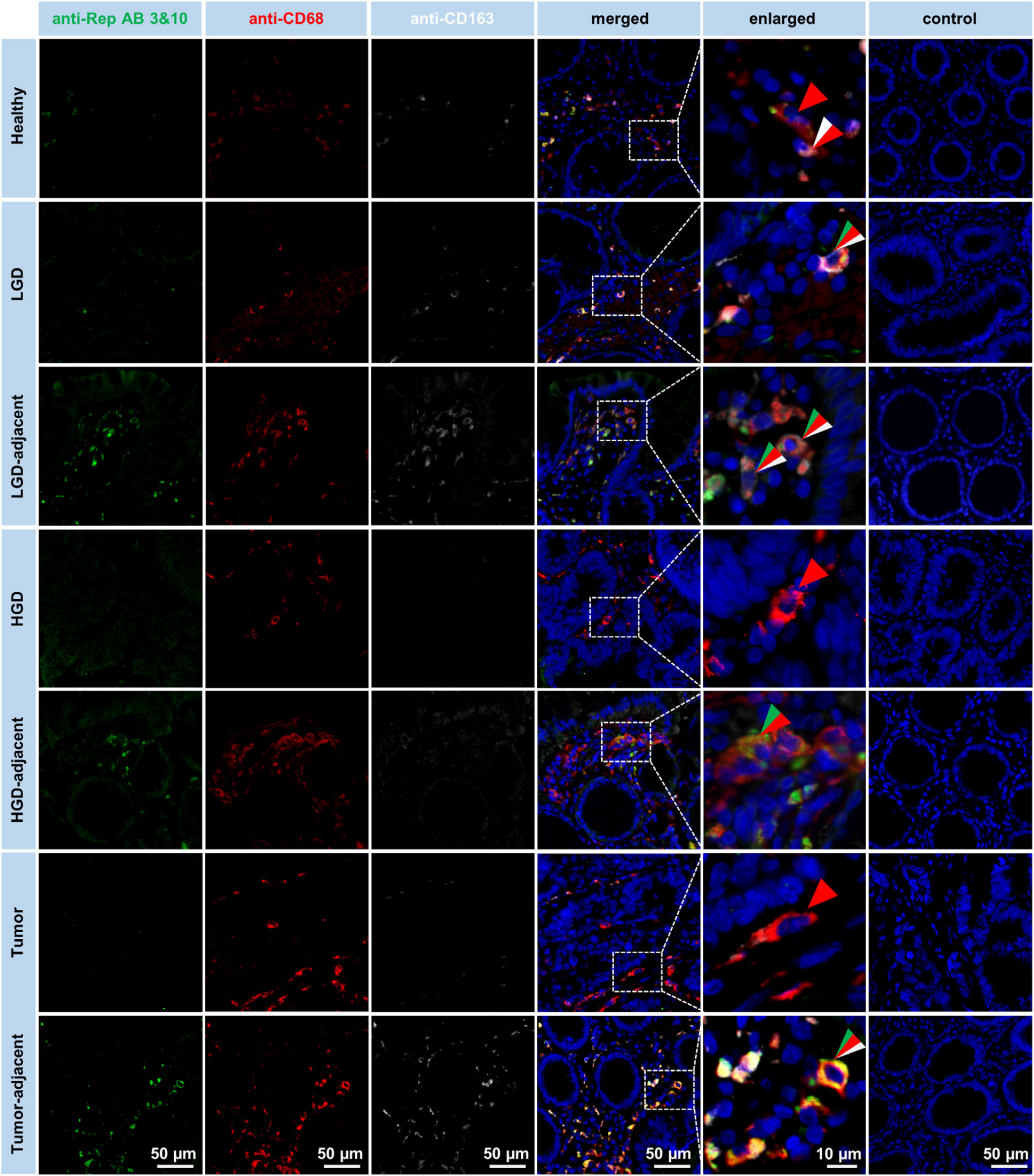
Co‐immunodetection of Rep/CD68/CD163 expression. Rep/CD68/CD163 co‐immunofluorescence in FFPE tissue sections prepared from the mucosa of healthy donors, from low‐/high‐grade dysplasia (LGD/HGD) and adjacent tissue and from CRC tumors and tumor‐adjacent tissues (paired). Co‐immunofluorescence microscopy shows cytoplasmic expression of Rep (green, green arrowheads), which is strongest in the tumor‐adjacent mucosa and is congruent with CD68 (red, red arrowheads) and CD163 (white, white arrowheads) expression in interstitial macrophages [scale bar: 50 μm, representative for each full column, DAPI staining of nuclei in blue, fivefold enlargements (scale bar: 10 μm, representative for the full column of enlargements) of indicated tissue parts and control staining performed with PBT diluent in combination with secondary detection antibodies].

**Fig. 2:**
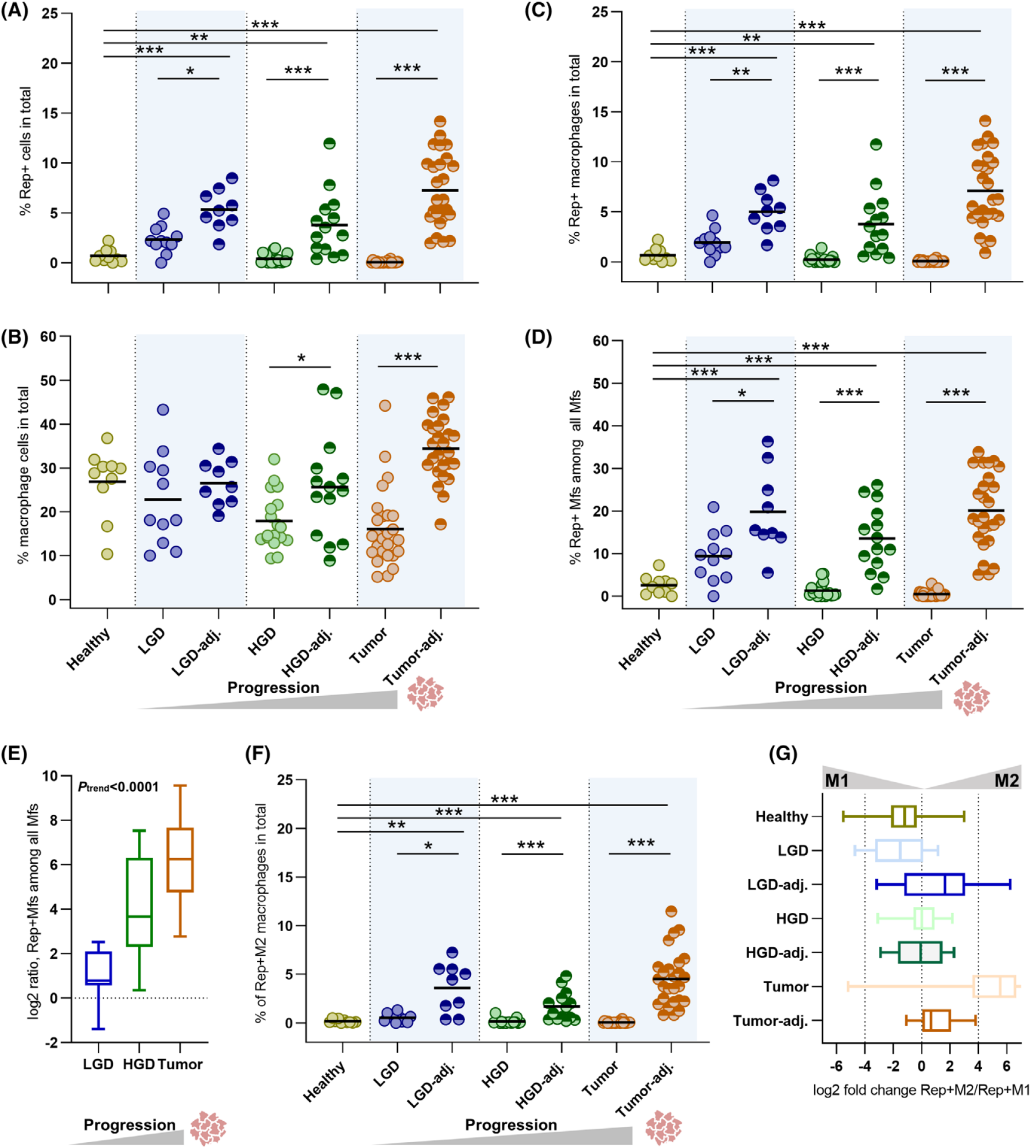
Quantification of Rep/CD68/CD163‐positive interstitial cells based on co‐immunofluorescence microscopy. Quantification of Rep+ interstitial cells (A) and CD68^+^ and/or CD163^+^ macrophages (Mfs) (B) is given in % of all interstitial cells. High levels of Rep+ Mfs (within all interstitial cells) (C) or within the population of Mfs (D) were observed in tissues adjacent to tumors and adjacent to LGD & HGD (for A–D: means illustrated as horizontal lines, full table of calculated *P*‐values in Table S2, Wilcoxon rank test for unpaired samples, Wilcoxon signed‐rank test for paired samples (dysplastic vs. adjacent) and *P*‐values adjusted by Holm correction). (E) The log2 ratio of adjacent vs. dysplastic detection of Rep+ Mfs increases from LGD over HGD to CRC (error bars indicating maximum/minimum, Jonckheere–Terpstra Test for trend: *P* < 0.0001). (F) Among Rep^+^ Mfs, the M2 phenotype was prevalent in the tissues adjacent to LGD, HGD and tumor (means illustrated as horizontal lines, Wilcoxon rank test for unpaired samples, Wilcoxon signed‐rank test for paired samples (dysplastic vs. adjacent) and *P*‐values adjusted by Holm correction). (G) This is also reflected by the increased log2‐fold ratio of Rep^+^ M2 vs. Rep^+^ M1 macrophages (error bars indicating maximum/minimum). Cohort sizes: healthy donors, *n* = 10; LGD – low‐grade dysplasia and adjacent tissue, *n* = 11 and *n* = 9, respectively; HGD – high‐grade dysplasia and adjacent tissue, *n* = 18 and *n* = 14, respectively; and CRC – colorectal cancer tumor and tumor‐adjacent tissues (paired), *n* = 26 for both. For log2‐fold‐based representations, detection events of exactly 0 were adjusted to half of the minimum of the respective cohort (frequency of corrections: 5× for Rep^+^ from all nuclei in CRC; 2× for Rep^+^ MF from all nuclei in HGD, 5× in CRC; and 2× for Rep^+^ MF within MF in HGD, 5× in CRC). Mf – macrophage, LGD – low‐grade dysplasia and HGD – high‐grade dysplasia. Significance: *P < 0.05, **P < 0.01 and ***P < 0.001.

We quantified Rep expression in a cohort of FFPE tissues containing paired tumor and tumor‐adjacent mucosa by histological (co‐) immunofluorescence staining and included a cohort of age‐matched controls (healthy colonic mucosa of tumor‐free individuals without inflammatory symptoms) for case‐versus‐control analysis (cohort descriptives in Table S1). In line with previous results [13], Rep expression was observed in the cytoplasm of interstitial macrophages of the lamina propria in the mucosa adjacent to tumors in CRC patients (Fig. 1). In addition, we performed cell‐based quantification of Rep expression and found that the number of Rep^+^ cells was significantly increased in the tumor‐adjacent mucosa of CRC patients, where on average 7% of interstitial cells were Rep^+^, when compared to the tumor (mean < 1%, *P* < 0.001) (Fig. 2A, summary of *P*‐values provided in Table S2). Notably, the mucosa of the age‐matched healthy donors showed significantly less Rep expression (mean < 1% Rep^+^ interstitial cells) when compared to the mucosa of CRC patients (mean 7%, *P* < 0.001).

### 3.2 Rep expression is increased in the adjacent tissues of dysplasia and tumors

Previous histological analysis has already shown Rep expression in the interstitium of adenomas/polyps [13]. Here, we expanded the histological analysis of Rep expression into low‐ and high‐grade dysplasia (LGD and HGD) including separate quantification of the unaffected adjacent mucosa (Fig. 1 and Fig. 2A). Rep expression in LGD (mean 2% Rep^+^ cells) was significantly increased compared to expression in healthy mucosa (mean < 1%, *P* = 0.031), while expression in HGD (< 1% mean) was at the level of the healthy controls (*P* = 0.514). Remarkably, also the adjacent tissues of LGD and HGD (mean 5% and 4% Rep^+^ cells, respectively) showed significantly increased Rep expression compared to the healthy controls (*P* = 0.001 and *P* = 0.006). Thus, we observed constantly high Rep expression for the adjacent mucosa of LGD, HGD and CRC, when compared to the mucosa of healthy controls. There is a significant decline in Rep expression in the dysplastic tissues from LGD over HGD to CRC; the latter showing almost a complete absence of Rep^+^ cells.

### 3.3 Rep expression is associated with an increased number of M2‐like macrophages

To analyze an association of Rep expression with the number of macrophages (Mfs), we quantified Rep cell‐based expression in combination with CD68 (as a general Mf marker) and CD163 (as a marker for the M2‐like Mf phenotype) (Fig. 2B–D). The total number of Mfs was highest in the tumor‐adjacent tissues of CRC patients, where on average 34% of interstitial cells showed staining (Fig. 2B). Slightly less Mfs were observed in the mucosa of the healthy controls (mean 27%) (*P* = 0.080 as compared to tumor adjacent). The distribution of Mfs in healthy samples ranged from a mean 10% to 37% and was higher and broader compared to the proportion of Rep^+^ cells in the same cohort (mean 0–2%).

In contrast, quantification of Rep‐expressing Mfs, again, showed similar trends as observed for the Rep expression alone (Fig. 2C). Rep^+^ Mfs were significantly increased in the mucosa of CRC patients (mean 7%) when compared to the healthy controls (< 1% on average, *P* < 0.001). Also, the adjacent tissues of LGD and HGD (mean 5% and 4%) as well as LGD (mean 2%) showed a higher number of Rep^+^ Mfs when compared to the healthy controls as well as HGD and tumor, where Rep^+^ Mfs were almost absent (all < 1% on average).

Within the population of Mfs, a fraction of around 20% was Rep^+^ in the mucosa of CRC patients and the adjacent tissue of LGD and HGD (mean 20% and 14%, respectively) (Fig. 2D). Around 9% of Mfs were Rep^+^ in the LGD, while on average only around 3% of Mfs were Rep^+^ in the healthy control, HGD and tumor samples. For the dysplastic tissues, there is a remarkable trend for reduction of Rep load in Mfs from LGD over HGD to tumor, which is reflected by an increased log2‐fold ratio of Rep^+^ Mfs in the interstitium versus dysplastic tissue parts (Fig. 2E).

Rep^+^ Mfs were further differentiated into an M1‐like (CD68^+^CD163^−^) or M2‐like phenotype (CD68^+/‐^CD163^+^). The Rep expression in the mucosa of CRC patients was associated with a CD163^+^ M2 phenotype (mean 4.5% of all interstitial cells, Fig. 2F) dominating over the M1 phenotype (mean 2.6% of all interstitial cells; the log2‐fold ratio M2/M1 is given in Fig. 2G). Comparable observations were made for the tissues adjacent to LGD (mean 3.6% M2 and 1.4% M1), while the situation was partly inversed for LGD (0.5% M2 and 1.4% M1) and HGD adjacent (0.1% M2 and 0.1% M1). In HGD, tumor and healthy controls, almost no Rep‐positive M2 Mfs were detected. The number of Rep‐negative M2 macrophages was equally high (around 6–10%, on average, of all interstitial cells) in healthy controls and the tissues adjacent to LGD, HGD and CRC (Fig. S2A; quantification of M1 and M2 Mfs irrespective of Rep is given in Fig. S2B,C, respectively).

### 3.4 Rep is expressed in the CRC tumor‐adjacent mucosa in tissue microarrays (TMA)

**Fig. 3:**
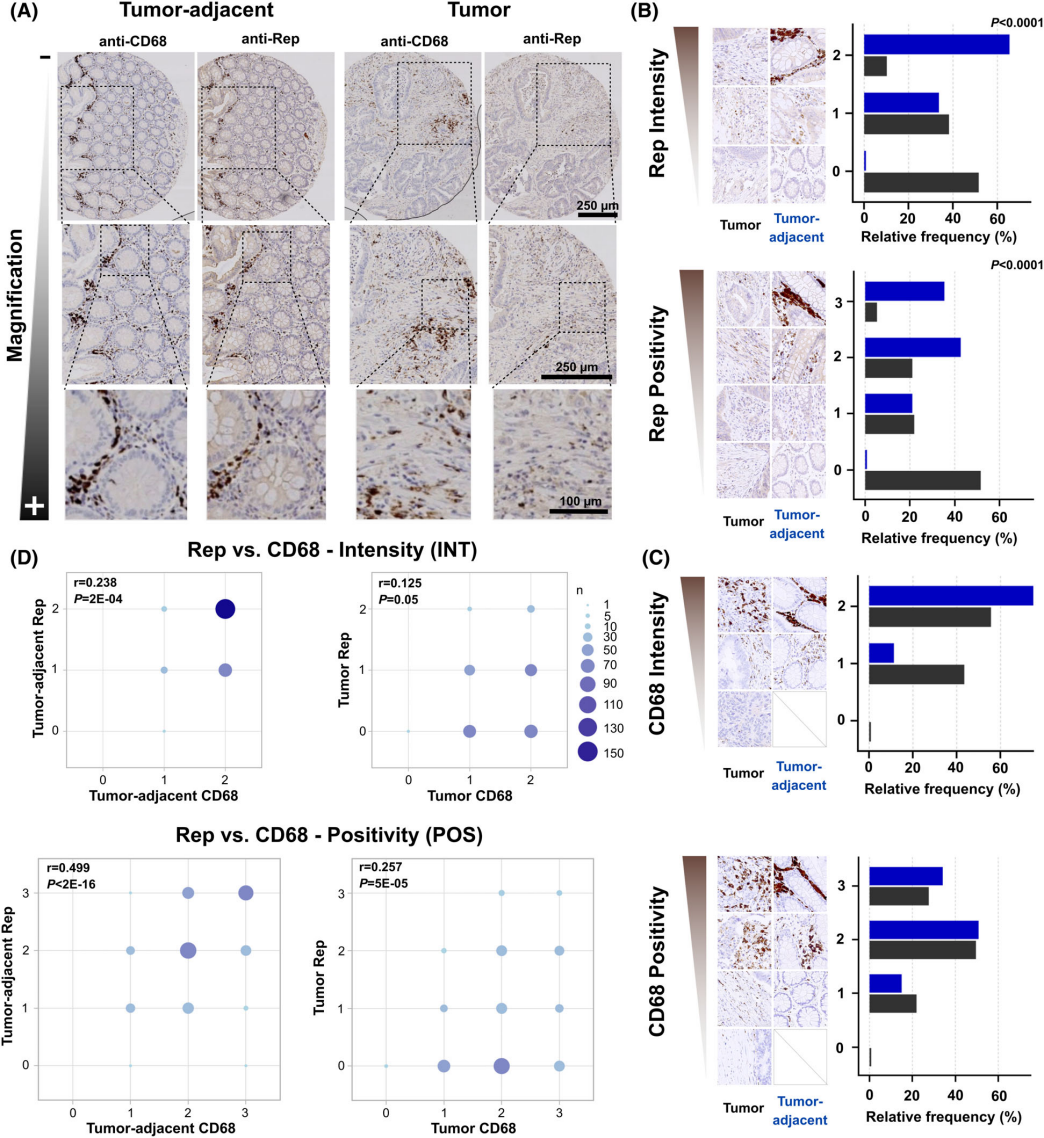
Quantification of Rep and CD68 expression based on CRC TMA. (A) Immunohistochemical Rep and CD68 macrophage staining for representative TMA tissue spots of the (paired) tumor‐adjacent mucosa (left panel) and tumor (right panel) of CRC patients [scale bar: 250 μm for the first two magnifications (upper and center rows) and 100 μm for the highest magnification (lower row)]. Distribution of Rep (B) and CD68 expression (C) in the paired tumor‐adjacent mucosa and tumor of CRC patients stratified in intensity (INT, scores 0–2) and spatial spread (POS, scores 0–3), including representative tissues stainings for each scoring (*n* = 246 individuals) (test for increased tumor‐adjacent vs. tumor Rep/CD68 POS and INT, respectively, by exact Wilcoxon–Pratt signed‐rank test). (D) Representation of Rep and CD68 INT and POS for tumor‐adjacent mucosa and tumor tissues (correlation of Rep and CD68 POS and INT tested by Spearman's rank correlation coefficient rho).

**Fig. 4:**
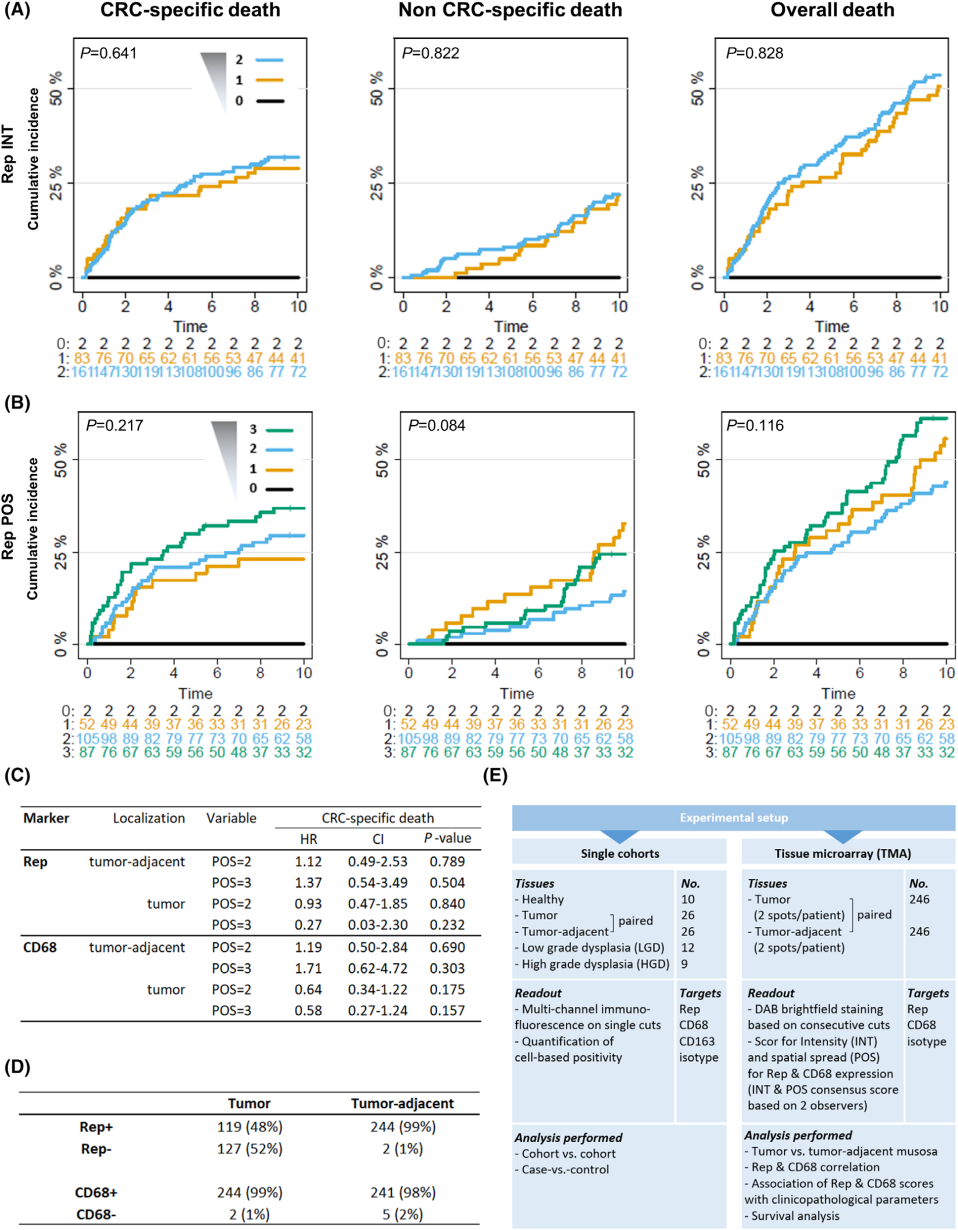
CRC TMA survival analysis and scoring. Individual cumulative incidence curves for intensity (INT, scores 0–2) (A) and spatial spread (POS, scores 0–3) of Rep staining (B) in the tumor‐adjacent mucosa for CRC‐specific, non‐CRC‐specific and overall death (including Gray (log‐rank type) tests for equality of cumulative incidence curves). (C) Hazard ratios (HR) including 95% confidence intervals (CI) and *P*‐values in cause‐specific multivariate proportional hazard models based on Rep and CD68 POS for the tumor‐adjacent mucosa or tumor accounting for clinical parameters. Cause‐specific multivariate proportional hazard models were adjusted for cancer stage and calculated based on linear age, ignoring BRAF or KRAS mutations due to unavailability and family history. Reference category for each of the CD68/Rep categories is POS “0/1”. (D) Summary of individual tumor or tumor‐adjacent mucosa patient tissues tested positive or negative for Rep (INT and/or POS > 0) or CD68 (INT and/or POS > 0). (E) Overview of the experimental setup for the cell‐based (IF) quantification of the single cohorts including clinical case‐vs.‐control cohorts and pretumor dysplasia (left) and for the CRC TMA cohort (right).

To analyze an association of Rep expression with clinic‐epidemiological parameters, we quantified the Rep (and CD68) expression in TMAs including tumor and paired tumor‐adjacent mucosa for each of the 246 patients with automated bright‐field IHC staining. Again, Rep expression was present in multiple foci in the lamina propria of the tumor‐adjacent mucosa without staining in the epithelial cells of the crypts of Lieberkühn (Fig. 3A, general setup of experimental staining and analysis provided in Fig. 4E). To quantify the antibody staining for each tissue spot, an immunoreactive score for intensity of the staining [intensity “INT” from 0 (min) to 2 (max)] and the size of the tissue area with positive staining [positivity “POS” from 0 (min) to 3 (max)] was assigned to each spot by two independent raters blinded for clinical data and final agreement “on screen” for one final INT/POS score (scoring examples included in Fig. 3B,C, scoring parameters given in Table S3, rater agreements on individual INT and POS scores shown in Table S4). A total of 244 of 246 examined mucosal tissue spots (99%) were positive for Rep detection (summarized in Fig. 4D) and showed strong staining of specific interstitial cells throughout large regions of the tissue. In contrast, in the tumor tissues, only 48% of the tissue spots (119 of 246) showed Rep expression, the majority of which localized in a smaller number of speckles with weaker staining. Isotype control staining did not show any staining in two representative TMAs of the DACHS cohort (*n* = 52 patients, in total) and > 20 different CRC tumor and non‐tumor tissues, which were repeatedly tested negative [13]. Significantly increased scores for Rep INT and POS were observed for the tumor‐adjacent mucosa when compared to tumor regions (*P* < 0.001 and P < 0.001, respectively, Fig. 3B, including scoring examples for INT and POS).

### 3.5 Rep expression correlates with the detection of CD68 ^+^ macrophages

CD68 staining was detected in 244 of 246 tumor tissues (99%) and 241 of 246 tumor‐ adjacent mucosa samples (98%) (Fig. 3A,C and Fig. 4D). In the tumor‐adjacent mucosa, congruent localization patterns of Rep and CD68 were observed. In the tumor spots, Rep detection was generally weaker and did not specifically correlate with the localization of CD68^+^ Mfs. An association between BMMF Rep and Mf detection levels was analyzed by correlation analysis of Rep and CD68 staining based on INT and POS and performed separately for both tumor‐adjacent mucosa and tumor tissues (Fig. 3D). In the tumor‐adjacent mucosa, an increased Rep INT was correlated with an increased CD68 INT (*r* = 0.236, *P* < 0.001). An even stronger correlation between Rep and CD68 scores was observed when analyzing staining POS (*r* = 0.499, *P* = < 0.001). In contrast, only a low level of correlation was observed for the tumor tissues for both INT (Pearson *r* = 0.125, *P* = 0.05) and POS (*r* = 0.257, *P* = < 0.001). Here, even tumor spots with high CD68 scores did not reveal specific detection of Rep.

### 3.6 Association between Rep detection and clinic‐epidemiological parameters

Possible associations of Rep & CD68 INT and POS with clinic‐epidemiological parameters such as age, sex, tumor localization and stage, microsatellite instability (MSI), therapy as well as limited categorized information on meat and milk consumption were analyzed (full distribution of Rep INT and POS scores provided in Tables S5 and S6 for tumor‐adjacent mucosa and Tables S7 and S8 for tumor tissues, including information on missing observations for the individual covariables). No statistically significant association between BMMF Rep (Table S9) detection as well as CD68 (Table S10) and any of the tested parameters could be observed after Holm adjustment for multiple testing, except for increased Rep INT in tumor‐adjacent mucosa in colon when compared to the rectum, which has to be interpreted with caution due to the limitation to only three categorization variables 0, 1 and 2 for INT (Table S9).

### 3.7 High Rep expression is associated with higher CRC‐specific death incidence

**Fig. 5:**
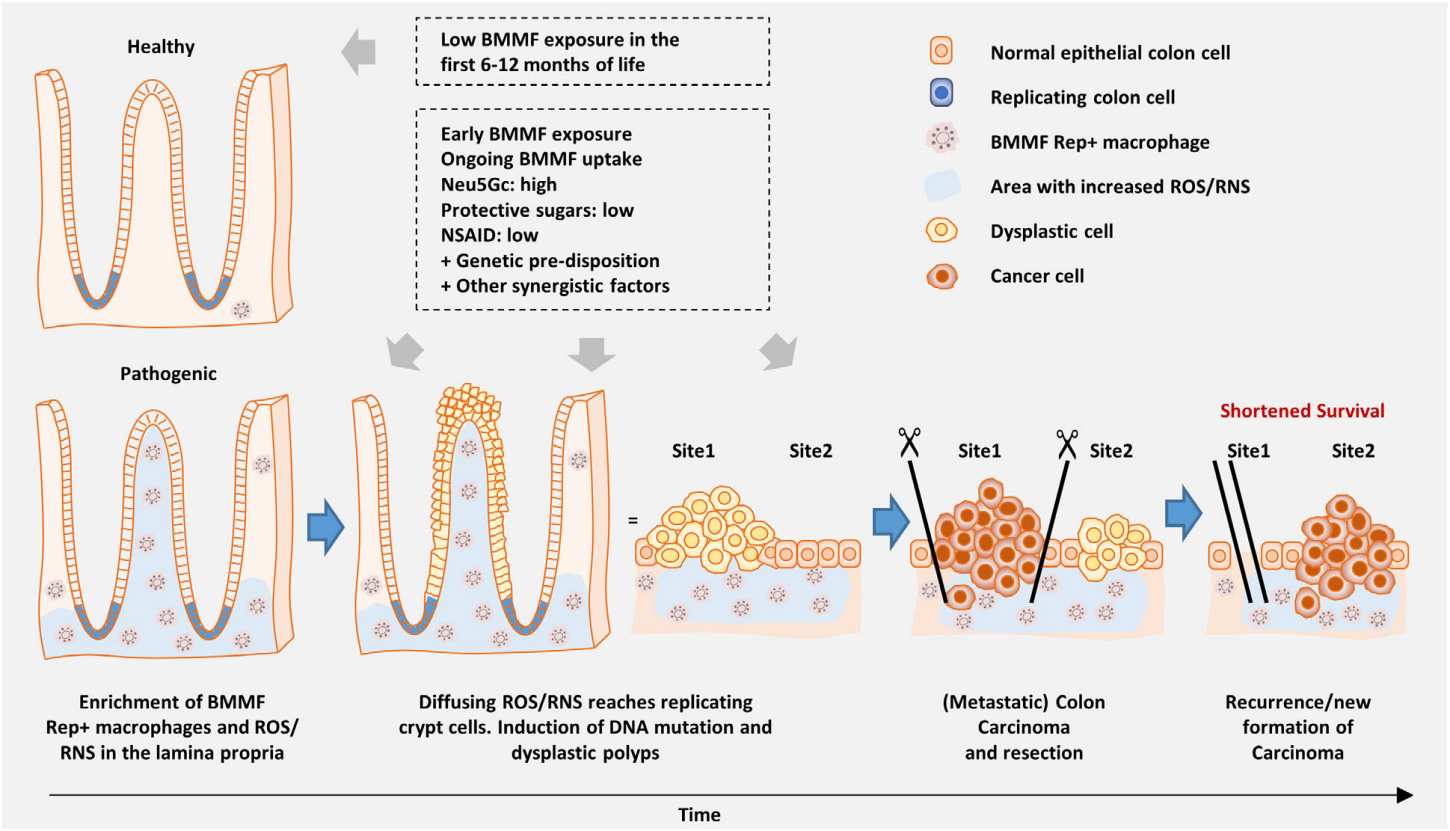
Model for BMMF as indirect drivers of CRC and risk factors for limited CRC‐specific survival (figure modified from [11] and [14]). The lack of an early encounter with BMMF, for example by prolonged breastfeeding in the first 6–12 months of life and avoiding bovine products results only in a low or moderate increase in BMMF Rep expression over time. In contrast, early encounter with BMMF causes an immunological condition with less efficient clearance of BMMF in the colonic/rectal mucosa (less resilience toward BMMF) and steadily increasing Rep expression levels. Consistently high BMMF exposure together with Neu5Gc uptake and low levels of protective sugars (such as the human milk oligosaccharides 2′−/3′‐fucosyllactose and lactotetraose) and additional synergistic contributions will result in persisting, high Rep expression in mucosal macrophages and increased chronic inflammation. This will increase the levels of diffusing reactive oxygen and nitrogen species (ROS/RNS) and contribute to DNA mutations in replicating crypt cells and induction of dysplasia and adenoma as precursors of CRC tumors. Even after resection of tumors, high mucosal Rep expression persists resulting in pro‐tumorigenic tissue conditions and ongoing induction of tumors/tumor recurrence and might also shape an environment promoting metastasis and therapy resistance.

In this exploratory analysis, cumulative incidence curves for CRC‐specific, non‐CRC‐specific and overall death were calculated to assess an association between Rep expression and patient survival (full overview of underlying Rep/CD68 INT and POS distributions stratified by outcome is given in Table S11). The individual incidence curves for CRC‐specific death were increased for higher Rep expression levels in the tumor‐adjacent tissues. The curve with highest Rep intensity (INT = 2) is located on top at highest death incidence and the curve for INT = 0 at the bottom (INT = 1 in between, Fig. 5A). A comparable, even more differentiated order of incidence curves was observed for Rep POS, with the incidence curve for highest Rep POS (POS = 3) on top (Fig. 5B). For non‐CRC‐specific death, no clear ordering of the individual incidence curves was observed. No clear order of the incidence curves was observed for the same type of analysis in tumor tissues (Fig. S3A).

Regarding CD68 expression, incidence curves for CD68 POS showed a clear ordering of incidence curves for CRC‐specific death, but in opposite directions for tumor and tumor‐adjacent tissues. In tumor tissues, curves with high CD68 POS are located at low incidence for CRC‐specific death (Fig. S3B, no clear order for non‐CRC‐specific death). In contrast, in tumor‐adjacent tissues, the curves with high CD68 POS located at high incidence for CRC‐specific death (Fig. S4, no clear order for non‐CRC‐specific death). Comparable tendencies were observed based on incidence curves of CD68 INT, but with less separation.

Cox proportional hazard models were performed with Rep and CD68 in a combined analysis but separate for INT and POS (full table of HRs given in Table S12). As expected, the cancer stage was significantly associated with decreased survival in all tested models and was accounted for in the calculation for the remaining test groups. In addition, male sex was associated with increased CRC‐specific survival when compared to female sex. All calculations related to Rep and CD68 were insignificant, thus only descriptive results can be given associated with high uncertainty. For increased Rep POS in tumor‐adjacent tissues, we identified HR > 1 (with category 2 or 3 vs. 0/1). For tumor Rep POS, we found HR < 1 comparing categories 2 and 3 versus 0/1. CD68 POS in tumor‐adjacent tissues revealed an HR > 1 when comparing categories 2 or 3 versus 1. In contrast, CD68 POS in the tumor was associated with an HR < 1 for comparison of categories 2 or 3 versus 0/1. Interpretation of HRs with respect to INT is more limited due to the lower number of categories (2 vs. 0/1). In contrast to CRC‐specific deaths, no monotonic relationships in case of non‐CRC deaths are found. Thus, after adjusting for covariates, similar observations as for univariate analysis in terms of cumulative incidence curves could be made.

## 4 Discussion

### 4.1 BMMF Rep expression in Mfs of the tumor‐adjacent mucosa is a marker for CRC

Rep IHC staining showed a strong Rep expression specifically in the tumor‐adjacent mucosa, both in the cohort of individual samples of paired tumor and tumor‐associated mucosal tissues and the TMA cohort of CRC patients. In the mucosa of CRC patients, BMMF Rep specifically localized in cytoplasmic areas with interstitial CD68^+^ Mfs in the lamina propria between the colonic crypts of Lieberkühn, which has also been shown before by using a broader set of Rep‐specific monoclonal antibodies including BMMF DNA isolation from positively stained tissue regions [6, 13]. The CD68^+^ Mfs represent a common type of myeloid immune cell in the interstitium promoting immunological defense under physiological conditions and might therefore be predisposed for a first encounter with BMMF [40]. In the tumor tissues, the inconclusive and weak Rep expression was not associated with CD68 staining and localized far from any neighboring nuclei. The low Rep expression in the tumor tissues and lack of Rep in the tumor cells is in line with a lack of BMMF DNA and protein detection observed for a previous cohort of CRC patients [6, 13].

Importantly, we observed a clear case‐versus‐control difference when comparing Rep expression levels in the peritumor mucosa of CRC patients with healthy age‐matched donors by cell‐based, quantitative analysis of Rep expression, which indicates that the increased Rep expression observed by BMMF Rep antibody staining might serve as histologic marker for CRC and supports a causative contribution of BMMF infection to CRC.

### 4.2 BMMF Rep expression correlates with CD68 ^+^ Mfs

If BMMFs contribute to cancer‐promoting chronic inflammation by direct or indirect interaction with Mfs, a correlation between Rep and CD68 Mf detection levels could exist. In co‐immunofluorescence analysis as well as in the analyses on consecutive cuts of the tumor‐adjacent mucosa of the TMA, the tissue regions showing high Rep expression were overlapping with the regions with high CD68 expression. In addition, in the TMA analyses, based on individual calculations of staining INT and POS, a higher Rep INT and POS in the tumor‐adjacent mucosa correlated with an increased CD68 INT and POS. We conclude that both CD68 and Rep antigens localized in the cytoplasmic compartment of Rep^+^/CD68^+^ double‐positive cells. This observation is in line with previous results showing co‐localization of Rep and CD68 antigens with co‐immunofluorescence staining in peritumoral CRC tissues [6, 13]. CD68 detection was generally stronger when compared to the Rep detection for all the cohorts including healthy controls, which is explainable by a generally high number of tissue‐resident Mfs in the colonic interstitium for the immunological defense of pathogens [40].

The question of whether Mfs specifically or non‐specifically take up BMMF nucleoprotein complexes (via specific receptors like sialic acid‐modified sugars) [11, 14] or take up BMMF DNA followed by production of BMMF protein in the macrophages cannot be answered and is currently being studied in follow‐up experiments.

### 4.3 Are Rep^+^ M2 macrophages the drivers of CRC?

The high number of Mfs observed even in the mucosa of the age‐matched healthy donors (including a population of Rep‐negative M2‐Mfs) indicates that the raw number of (M2) Mfs *per se* might not be a negative risk factor for CRC (as also indicated by the missing correlation of CD68 expression and CRC survival in this descriptive study). However, the number of Rep^+^ (M2) Mfs shows a clear case‐versus‐control difference and also is constantly increased in LGD, HGD and HGD‐adjacent tissues. Thus, Rep, but not CD68, expression might be regarded as potentially causative and important hallmark for induction of polyps and CRC. A differentiation toward an M2 phenotype was observed for Rep^+^ Mfs in the tissues adjacent to LGD, adjacent to HGD and adjacent to tumor (less Rep^−^ M2 Mfs in LGD and HGD). Healthy controls had a larger fraction of (Rep^−^) M1 Mfs, which might be indicative of an active immunity against BMMF with efficient clearance of BMMF infections over time.

Indeed, this general situation of case‐versus‐control sensitive BMMF–macrophage cell populations would reflect the induction of cancer based on a histiocytic, inflammatory‐driven, indirect mechanism affecting large areas of normal tissue regions over several decades [11, 14, 16, 34]. The situation might be comparable to the indirect carcinogenesis outlined for Hepatitis C (hepatocellular carcinoma) [41], HIV 1 and 2 (contributing to several cancer types) [42, 43] and *Helicobacter pylori* infection (gastric and stomach cancer) [44], where the infectious agent does not persist in the induced cancer cells and where the malignant growth propagates independently of the infectious agent but coincides with chronic inflammatory reaction in the affected tissues [11, 14, 16]. In our previously proposed hypothesis of indirect BMMF carcinogenesis, the infectious risk factors are not present and do not persist in the tumor cells. Instead, upon interstitial infection, they induce pathogenic modulations over long‐latency periods including chronic inflammation and induction of radicals diffusing over long distances [11]. The radicals eventually also reach replication‐competent stem and early daughter cells in the colonic crypts (which themselves are BMMF negative and were never in contact with BMMF), where they cause random DNA mutations. For a small number of cells, specific cancer‐driver mutations might be fixed over time allowing development into adenoma and polyps as precursors for CRC [11, 13]. This situation explains the lack of Rep detection not only in the tumor cells but also in the stroma, which underwent massive changes during tumor progression, and at the same time, explains the pronounced Rep expression in the tumor surrounding mucosa.

### 4.4 Association of BMMF expression with clinic‐epidemiological parameters

Because of the limited size of the TMA cohort lacking any healthy controls (risk to focus on the generally high BMMF exposure expected for this cohort of CRC patients) as well as poor categorization, especially for the dietary parameters describing the young age meat and milk consumption (e.g. the study included no donors without meat consumption), the lack of a statistically significant association of the Rep and/or CD68 expression with meat or milk consumption was largely anticipated. Dedicated and appropriately powered larger databases are needed to study this relevant question.

### 4.5 BMMF‐based evaluation of patient survival (prognosis)

In this descriptive study, the quantification of tumor‐adjacent Rep expression based on staining INT and POS showed that incidence curves with increased Rep expression localized at higher incidence when compared to the curves with low Rep expression for CRC‐specific death (no specific order in non‐CRC death supporting the specificity of the observation). This observation is in line with HR > 1 in Cox proportion hazard models, albeit no analysis for Rep and CD68 showed significance, which limits the general interpretation of Rep (and CD68) expression on the prognosis of CRC patients. CD68 expression in the peritumor showed an association with a less favorable prognosis while tumor CD68 expression was linked with a beneficial prognosis, which was reported in serval studies [45].

While tumor induction itself can be explained by indirect BMMF‐ and chronic inflammation‐driven carcinogenesis [11, 13, 14, 16], the question is why high mucosal Rep expression would negatively affect prognosis and survival of CRC patients, whose tumors were surgically removed. An explanation might be that increased Rep expression modulates the tumor microenvironment in an unfavorable way, promoting tumor recurrence and metastasis (Fig. 5). Following this rationale, high Rep expression might shape the tumor‐adjacent mucosa of cancer patients (also after tumor resection) in such a way that an unfavorable immunological state with a worse prognosis is induced, for example by induction of higher levels of pro‐tumorigenic M2 macrophages (and/or protumorigenic, regulatory T‐cells). Under these conditions, individuals with a shorter CRC‐specific or all‐cause survival might clear BMMF expression less effectively from the mucosa when compared to individuals with longer survival and suffer from continuingly high chronic inflammation, tumor recurrence, metastasis and therapy resistance.

### 4.6 BMMF‐induced inflammation and BMMF biomarkers

BMMF Rep expression in the tumor‐adjacent mucosa of CRC patients was observed for 99% of patients (TMA cohort) and was linked to Rep^+^ M2 CD68^+^ interstitial Mfs (IF analyses), which were almost completely absent in mucosal tissues of non‐CRC healthy controls. We additionally showed in this descriptive study that high BMMF Rep expression might be indicative of (early) dysplasia which increases the risk of CRC as progenitors for cancer. This supports a potentially causative involvement of BMMF in CRC. The interpretation of a prognostic value of BMMF detection concerning patient survival must be deciphered individually from any causative involvements. The indication of poor CRR‐specific and overall patient survival with high Rep positivity (POS) was visible but not significant, which limits the power of interpretation and requires further analyses.

### 4.7 Limitations of this study and future perspectives

The limitations of the TMA cohort did not allow a more specific analysis of an association of Rep (CD68) expression with clinic‐epidemiological parameters such as meat and milk consumption. The most obvious limitations were inadequate categorization (ignoring red meat and exposure at an early age) and a lack of healthy individuals, which might generally have a lower BMMF exposure than CRC patients. The study is also limited by the use of only two tissue spots per patient, albeit larger metric analyses of mucosal tissues taken at variable distances from the primary tumor generally showed a high Rep expression independently from the distance to the tumor. Future studies are needed to analyze an association of BMMF expression with the metastatic state not assessed here.

Future test cohorts should be recruited from different geographical regions to assess the applicability of the present Rep antibody and the histological scoring. This is of particular importance because we currently know more than 20 isolates belonging to the same group of BMMF isolates as H1MSB.1 [5, 6], whose Rep was used for production of AB3 and AB10 and which might have a different regional (global) distribution and comprise different Reps, which might be only partly detected with the present Rep antibodies [6, 13]. Also, additional BMMF gene products might be relevant as disease markers. Future studies should also test a possible effect of non‐steroidal anti‐inflammatory drugs (NSAIDs) or metformin, which were reported to reduce the incidence of several cancer types, on the Rep expression, and other therapies which might help to identify preventive or therapeutic routes for BMMF intervention in future [11, 14, 46].

## 5 Conclusion

BMMF Rep expression was localized primarily in M2‐like interstitial macrophages of the tumor‐adjacent mucosa of CRC patients, while significantly less Rep expression was observed in the tumor and healthy controls, the latter underlining an association of BMMF with CRC. Despite not being statistically significant, the link of high tumor‐adjacent Rep expression with increased CRC‐specific death suggests a contribution of BMMF on patient prognosis even after tumor resection. Monitoring of Rep expression in biopsies might help to not only understand BMMF‐specific induction of CRC but also identify individuals at (early) risk for CRC and might serve to evaluate therapy response. Early BMMF detection might offer options for BMMF‐specific preventive or therapeutic intervention.

## Conflict of interest

The authors declare no conflict of interest.

## Author contributions

TB, AB‐K and EN designed the study. TB, AB‐K, EN and MW wrote the manuscript. MHE, EA, MHO, AK‐S and HB revised the manuscript. DH and CE performed immunohistochemistry staining. JC‐C, HB, MHO, EA, PS‐K, EH, AB and MvW coordinated the clinical studies, selected and provided the tissue samples. AB, EH and MvW classified the tissue samples. CT generated monoclonal antibodies. EN, AB‐K and TB performed immunohistochemical quantification. EN, AB‐K, MW, TB, AK‐S, MHO, HB and DK performed data analysis. MW and AK‐S performed statistical analysis. MHE, JC‐C, EA and PS‐K were involved in data interpretation. All authors contributed to the drafting of the manuscript and approved the final version of the article including the authorship list.

### Peer review

The peer review history for this article is available at https://publons.com/publon/10.1002/1878‐0261.13390.

## Supporting information


**Fig. S1.** Detection of overexpressed H1MSB.1 replication protein (Rep) with anti‐Rep antibodies (AB) AB3 and AB10 based on western blotting (WB) and immunohistochemistry (IHC).
**Fig. S2.** Cell‐based quantification of macrophages after Rep/CD68/CD163 co‐immunofluorescence microscopy.
**Fig. S3.** Cumulative death incidence for tumor Rep (A) and CD68 (B) intensity (INT) and spread of the staining (POS) for CRC, non‐CRC and overall death.
**Fig. S4.** Cumulative death incidence for CD68 intensity (INT) and spread of the staining (POS) in the tumor‐adjacent mucosa for CRC, non‐CRC and overall death.
**Table S1.** Information on tissues acquired from healthy donors and patients with low‐grade dysplasia (LGD), high‐grade dysplasia (HGD) and CRC.
**Table S2.** *P*‐value summary for comparison of cell‐based Rep/CD68/CD163 expression.
**Table S3.** Scoring parameters for quantification of Rep and CD68 expression in the CRC TMA based on an immunoreactive score for staining intensity (INT) and spatial spread of the staining (POS) after initial, independent scoring by two individual judges.
**Table S4.** Agreement of the Rep and CD68 staining intensity (INT) (A) and Rep and CD68 spread of the staining (POS) (B) by two independent raters.
**Table S5.** Distributions of clinical parameters stratified by the Rep staining intensity (INT) in the tumor‐adjacent mucosa.
**Table S6.** Distributions of clinical parameters stratified by the spread of the Rep staining (POS) in the tumor‐adjacent mucosa.
**Table S7.** Distributions of clinical parameters stratified by the Rep staining intensity (INT) in tumor tissues.
**Table S8.** Distributions of clinical parameters stratified by the spread of the Rep staining (POS) in tumor tissue.
**Table S9.** Association of Rep staining intensity (INT) and spread on the staining (POS) in tumor and tumor‐adjacent mucosa of CRC patients with clinicopathological parameters.
**Table S10.** Association of CD68 staining intensity (INT) and spread of the staining (POS) in tumor and tumor‐adjacent mucosa of CRC patients with clinicopathological parameters.
**Table S11.** Distribution of Rep and CD68 staining intensity (INT) (A) and spread of the staining (POS) (B) stratified by censored (n0), CRC‐specific death (n_CRC_), non‐CRC‐specific death (n_other_) and unstratified (n_all_).
**Table S12.** Cause‐specific proportional hazard models combined for Rep and CD68 for CRC‐specific and non‐CRC‐specific death.

## Data Availability

The data that support the findings of this study are available from the corresponding author upon request.
