# Supplementary material for: Bovine meat and milk factor protein expression in tumor‐free mucosa of colorectal cancer patients coincides with macrophages and might interfere with patient survival
Source: Mol Oncol. 2023 Feb 22;18(5):1076–92. doi: 10.1002/1878-0261.13390 (PMC11076986; doi:10.1002/1878-0261.13390)
Supplement: Supplementary file 1 — Fig. S1. Detection of overexpressed H1MSB.1 replication protein (Rep) with anti‐Rep antibodies (AB) AB3 and AB10 based on western blotting (WB) and immunohistochemistry (IHC). Fig. S2. Cell‐based quantification of macrophages after Rep/CD68/CD163 co‐immunofluorescence microscopy. Fig. S3. Cumulative death incidence for tumor Rep (A) and CD68 (B) intensity (INT) and spread of the staining (POS) for CRC, non‐CRC and overall death. Fig. S4. Cumulative death incidence for CD68 intensity (INT) and spread of the staining (POS) in the tumor‐adjacent mucosa for CRC, non‐CRC and overall death. Table S1. Information on tissues acquired from healthy donors and patients with low‐grade dysplasia (LGD), high‐grade dysplasia (HGD) and CRC. Table S2. P‐value summary for comparison of cell‐based Rep/CD68/CD163 expression. Table S3. Scoring parameters for quantification of Rep and CD68 expression in the CRC TMA based on an immunoreactive score for staining intensity (INT) and spatial spread of the staining (POS) after initial, independent scoring by two individual judges. Table S4. Agreement of the Rep and CD68 staining intensity (INT) (A) and Rep and CD68 spread of the staining (POS) (B) by two independent raters. Table S5. Distributions of clinical parameters stratified by the Rep staining intensity (INT) in the tumor‐adjacent mucosa. Table S6. Distributions of clinical parameters stratified by the spread of the Rep staining (POS) in the tumor‐adjacent mucosa. Table S7. Distributions of clinical parameters stratified by the Rep staining intensity (INT) in tumor tissues. Table S8. Distributions of clinical parameters stratified by the spread of the Rep staining (POS) in tumor tissue. Table S9. Association of Rep staining intensity (INT) and spread on the staining (POS) in tumor and tumor‐adjacent mucosa of CRC patients with clinicopathological parameters. Table S10. Association of CD68 staining intensity (INT) and spread of the staining (POS) in tumor and tumor‐adjacent mucosa of CR [file MOL2-18-1076-s001.pdf]

Bovine meat and milk factor protein expression in tumor-free mucosa of colorectal cancer patients coincides with macrophages and might interfere with patient survival

Ekaterina Nikitina, Amelie Burk-Körner, Manuel Wiesenfarth, Elizabeth Alwers, Danijela Heide, Claudia Tessmer, Claudia Ernst, Damir Krunic, Petra Schrotz-King, Jenny Chang-Claude, Moritz von Winterfeld, Esther Herpel, Alexander Brobeil, Hermann Brenner, Mathias Heikenwalder, Michael Hoffmeister, Annette Kopp-Schneider, Timo Bund

SUPPLEMENTARY MATERIAL

Table of contents:

Supplementary Figures S1-S4
Supplementary Tables S1-S12
References

Supplementary Figures S1-S4

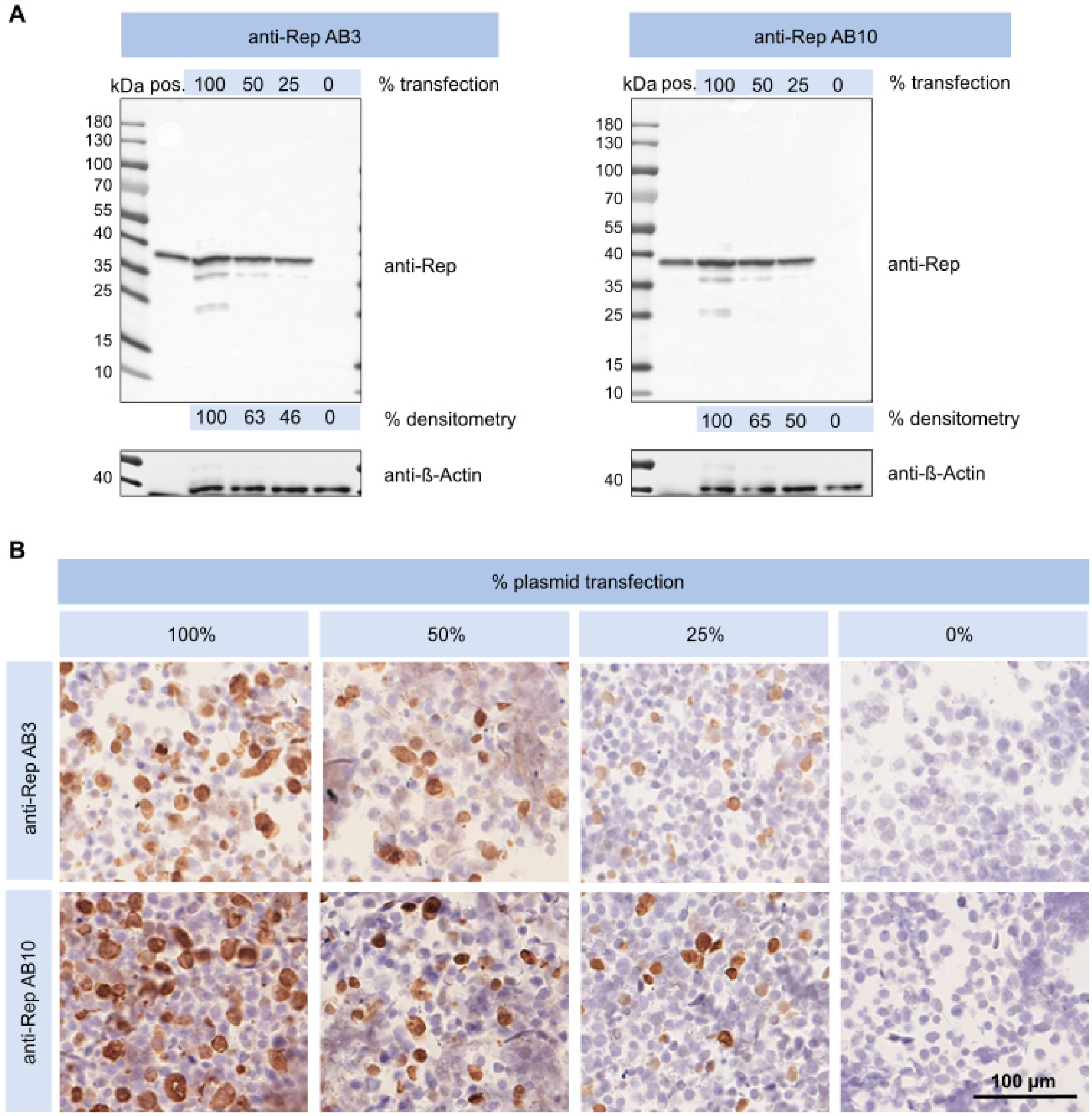

Fig. S1: Detection of overexpressed H1MSB.1 replication protein (Rep) with anti Rep antibodies (AB) AB3 and AB10 based on Western Blotting (WB) and immunohistochemistry (IHC) [1]. H1MSB.1 Rep was overexpressed in HEK293TT cells after transient DNA transfection (100, 50, 25 or 0% transfected DNA). **(A)** After 72 h, cells were analyzed by WB (Rep target band (37 kDa) detected with AB3 and AB10). **(B)** An aliquot of cells was detached, pelleted and paraffin-embedded prior to anti-Rep DAB IHC. A dose-dependent detection of the Rep antigen was observed for AB3 and AB10 (Figure taken from [1]).

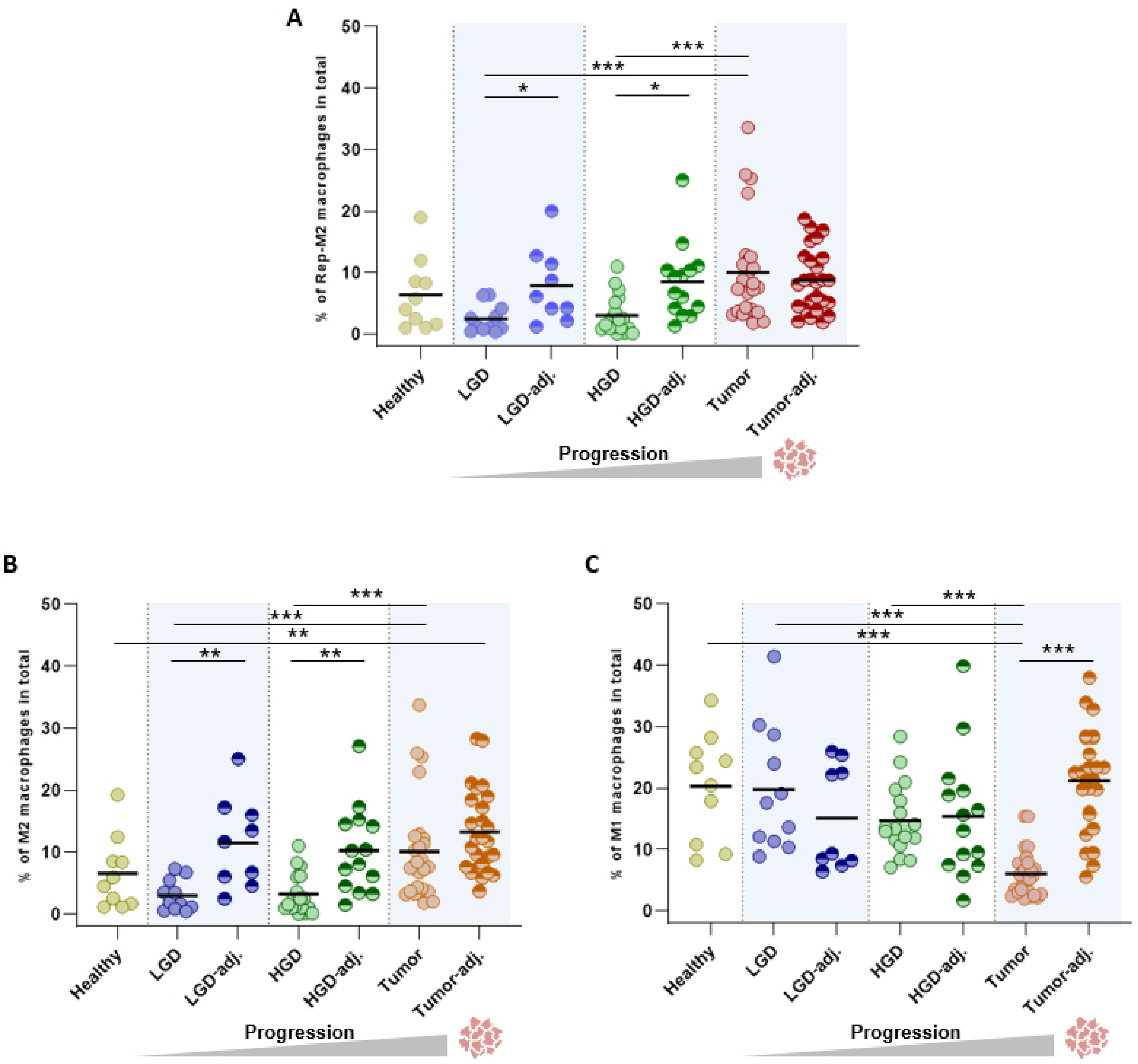

Fig. S2: Cell-based quantification of macrophages after Rep/CD68/CD163 co-immunofluorescence microscopy. **(A)** Quantification of Rep-negative M2 macrophages (Rep$^-$/CD68$^{+/-}$/CD163$^+$). Quantification of CD68$^{+/-}$/CD163$^+$ M2 macrophages **(B)** and CD68$^+$/CD163$^-$ M1 macrophages **(C).** Means illustrated as horizontal lines, Wilcoxon rank test for unpaired samples, Wilcoxon signed rank test for paired samples (dysplastic vs. adjacent), *P*-values adjusted by Holm correction, LGD – low grade dysplasia, HGD – high grade dysplasia. Significance: **P*<0.05, ***P*<0.01, ****P*<0.001.

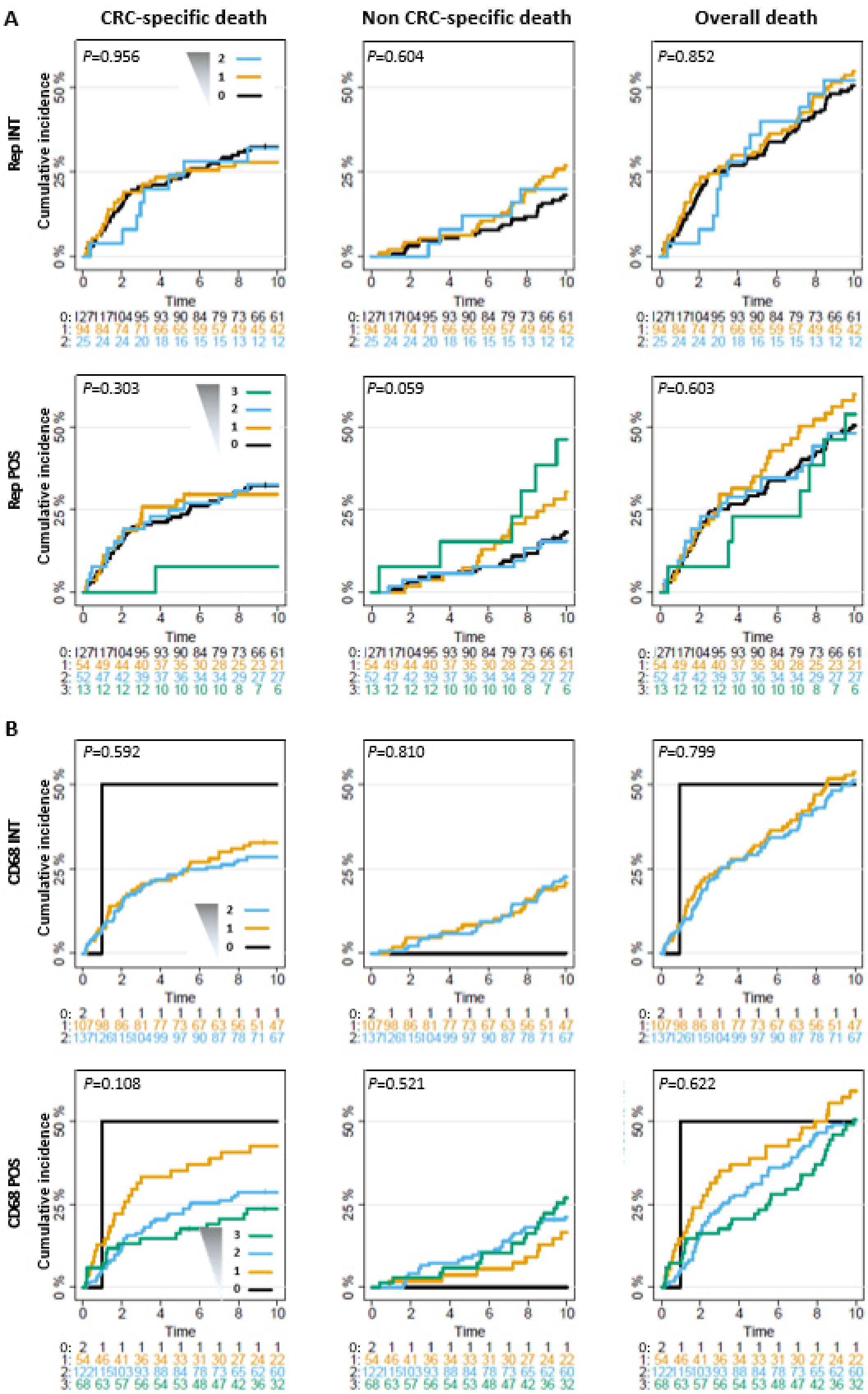

Fig. S3: Cumulative death incidence for tumor Rep **(A)** and CD68 **(B)** intensity (INT) and spread of the staining (POS) for CRC, non-CRC, and overall death (including Gray (log-rank type) tests for equality of cumulative incidence curves).

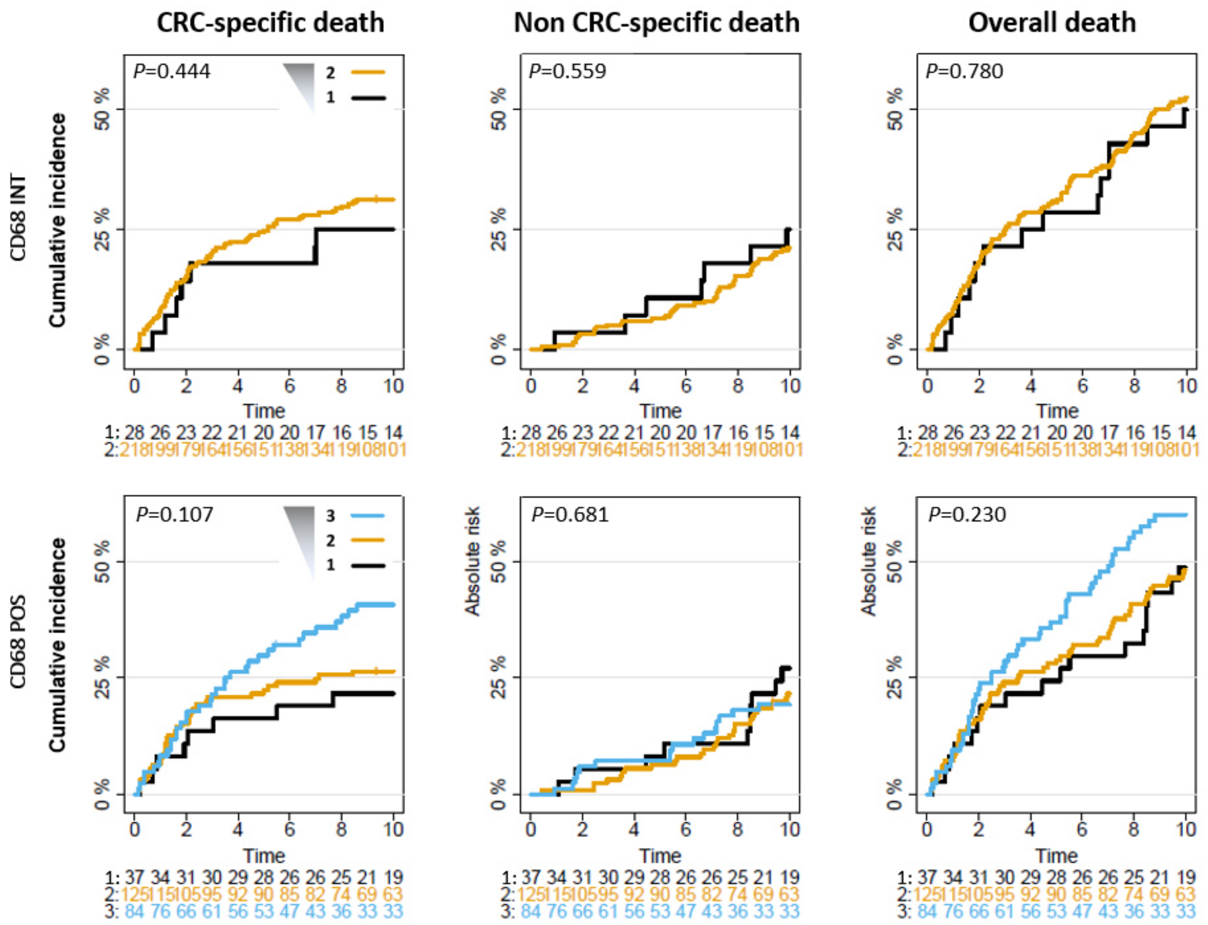

Fig. S4: Cumulative death incidence for CD68 intensity (INT) and spread of the staining (POS) in the tumor-adjacent mucosa for CRC, non-CRC, and overall death (including Gray (log-rank type) tests for equality of cumulative incidence curves).

Supplementary Tables S1-S12

Variable	Levels	nhealthy	%healthy	nLGD	%LGD	nHGD	%HGD	nCRC	%CRC	nall	%all
age	37-55	4	40.0	5	45.5	3	16.6	15	57.6	27	41.5
	56-65	4	40.0	3	27.3	4	22.2	4	15.3	15	23.1
	66-79	2	20.0	1	9.1	9	50.0	7	26.9	19	29.2
	80-89	0	0.0	2	18.2	2	11.1	0	0.0	4	6.2
	all	10	100.0	11	100.0	18	100.0	26	100.0	65	100.0
sex	female	5	50.0	5	45.4	11	61.1	14	53.8	35	53.8
	male	5	50.0	6	54.5	7	38.9	12	46.2	30	46.2
	all	10	100.0	11	100.0	18	100.0	26	100.0	65	100.0
localization	right colon	3	30.0	0	0.0	0	0.0	6	23.0	9	13.8
	left colon	3	30.0	0	0.0	0	0.0	9	34.6	12	18.5
	rectum	2	20.0	0	0.0	0	0.0	10	38.4	12	18.5
	missing	2	0.0	11	100.0	18	100.0	1	3.8	32	49.2
	all	10	100.0	11	100.0	18	100.0	26	100.0	65	100.0
tumor stage	1	0	0.0	0	0.0	0	0.0	0	0.0	0	0.0
	2	0	0.0	0	0.0	0	0.0	4	15.4	4	15.4
	3	0	0.0	0	0.0	0	0.0	16	61.5	16	61.5
	4	0	0.0	0	0.0	0	0.0	5	19.2	5	19.2
	missing	0	0.0	0	0.0	0	0.0	1	3.8	1	3.8
	all	0	0.0	0	0.0	0	0.0	26	100.0	26	100.0
node status	N0	0	0.0	0	0.0	0	0.0	9	34.6	9	34.6
	N1-2	0	0.0	0	0.0	0	0.0	16	61.5	16	61.5
	missing	0	0.0	0	0.0	0	0.0	1	3.8	1	3.8
	all	0	0	0	0	0	0	26	100	26	100.0
metastasis status	M0	0	0.0	0	0.0	0	0.0	18	69.2	18	69.2
	M1-2	0	0.0	0	0.0	0	0.0	7	26.9	7	26.9
	missing	0	0.0	0	0.0	0	0.0	1	3.8	1	3.8
	all	0	0.0	0	0.0	0	0.0	26	100.0	26	100.0
neoadj. chemotherapy	yes	0	0.0	0	0.0	0	0.0	4	15.4	4	15.4
	no	0	0.0	0	0.0	0	0.0	21	80.7	21	80.8
	missing	0	0.0	0	0.0	0	0.0	1	3.8	1	3.8
	all	0	0.0	0	0.0	0	0.0	26	100.0	26	100.0

Table S1: Information on tissues acquired from healthy donors and patients with low grade dysplasia (LGD), high grade dysplasia (HGD) and CRC.

% Macrophages in total (upper triangle) / % Rep cells in total (lower triangle)

	Healthy	LGD	LGD-adj.	HGD	HGD-adj.	Tumor	Tumor-adj.	
Healthy		1.000	1.000	0.080	1.000	**0.042**	0.080	Healthy
LGD	**0.031**		0.359	0.476		0.243		LGD
LGD-adj.	**0.001**	**0.012**			0.734		**0.011**	LGD-adj.
HGD	0.514	**0.000**			**0.041**	0.476		HGD
HGD-adj.	**0.006**		0.191	**0.000**			**0.011**	HGD-adj.
Tumor	**0.002**	**0.000**		**0.002**			**0.000**	Tumor
Tumor-adj.	**0.000**		0.191		**0.014**	**0.000**		Tumor-adj.
	Healthy	LGD	LGD-adj.	HGD	HGD-adj.	Tumor	Tumor-adj.	

% Rep cells in total

% Rep+ macrophages in total macrophages (upper triangle) / % Rep+ macrophages in total (lower triangle)

	Healthy	LGD	LGD-adj.	HGD	HGD-adj.	Tumor	Tumor-adj.	
Healthy		**0.017**	**0.001**	0.320	**0.001**	**0.008**	**0.000**	Healthy
LGD	**0.038**		**0.027**	**0.000**		**0.000**		LGD
LGD-adj.	**0.001**	**0.008**			0.282		0.838	LGD-adj.
HGD	0.190	**0.000**			**0.000**	0.064		HGD
HGD-adj.	**0.006**		0.256	**0.000**			0.093	HGD-adj.
Tumor	**0.002**	**0.000**		**0.038**			**0.000**	Tumor
Tumor-adj.	**0.000**		0.256		**0.014**	**0.000**		Tumor-adj.
	Healthy	LGD	LGD-adj.	HGD	HGD-adj.	Tumor	Tumor-adj.	

% Rep+ macrophages in total

Table S2: *P*-value summary for comparison of cell-based Rep/CD68/CD163 expression. *P*-values for comparison of cohorts based on Rep$^+$ cells and macrophages among all interstitial cells (left) and Rep$^+$ macrophages among all interstitial cells and Rep$^+$ macrophages within the population of macrophages (right). Wilcoxon rank sum test used for unpaired samples. Wilcoxon signed rank test used for paired samples (dysplastic vs. adjacent). *P*-values adjusted for multiplicity by using Holm correction separately by outcome variable and groups of tests, i.e. adjustment within group “healthy vs. all other groups”, “all pairs of dysplastic LGD, HGD, tumor”, “all pairs of LGD-adjacent, HGD-adjacent, tumor-adjacent” and group “paired samples dysplastic vs adjacent”. *P*-values below 0.05 are highlighted. LGD – low grade dysplasia, HGD – high grade dysplasia.

Target	Intensity (Staining intensity, INT)	INT	Positivity (proportion of positive cells, POS)	POS
**Rep**	0	no detection	0	0
	1	moderate	1	1-10%
	2	strong	2	11-30%
			3	>31%
**CD68**	0	no detection	0	0
	1	moderate	1	<20%
	2	strong	2	20-60%
			3	>60%

Table S3: Scoring parameters for quantification of Rep and CD68 expression in the CRC TMA based on an immunoreactive score for staining intensity (INT) and spatial spread of the staining (POS) after initial, independent scoring by two individual judges (scoring agreement shown in Table S4).

A

	Rep INT	n differing scoring	% differing scoring	n equal scoring	% equal scoring	n all	% all
Tumor	0	4	3.1	123	96.9	127	51.6
	1	48	51.1	46	48.9	94	38.2
	2	22	88.0	3	12.0	25	10.2
	all	74	30.1	172	69.9	246	100.0
Tumor-adjacent	0	0	0.0	2	100.0	2	0.8
	1	71	85.5	12	14.5	83	33.7
	2	78	48.4	83	51.6	161	65.4
	all	149	60.6	97	39.4	246	100.0

	CD68 INT	n differing scoring	% differing scoring	n equal scoring	% equal scoring	n all	%all
Tumor	0	1	50.0	1	50.0	2	0.8
	1	88	82.2	19	17.8	107	43.5
	2	82	59.9	55	40.1	137	55.7
	all	171	69.5	75	30.5	246	100.0
Tumor-adjacent	0	0	-	0	-	0	0.0
	1	27	96.4	1	3.6	28	11.4
	2	163	74.8	55	25.2	218	88.6
	all	190	77.2	56	22.8	246	100.0

B

	Rep POS	n differing scoring	% differing scoring	n equal scoring	% equal scoring	n all	%all
Tumor	0	4	3.1	123	96.9	127	51.6
	1	21	38.9	33	61.1	54	22.0
	2	38	73.1	14	26.9	52	21.1
	3	11	84.6	2	15.4	13	5.3
	all	74	30.1	172	69.9	246	100.0
Tumor-adjacent	0	0	0.0	2	100.0	2	0.8
	1	45	86.5	7	13.5	52	21.1
	2	70	66.7	35	33.3	105	42.7
	3	34	39.1	53	60.9	87	35.4
	all	149	60.6	97	39.4	246	100.0

	CD68 POS	n differing scoring	% differing scoring	n equal scoring	% equal scoring	n all	%all
Tumor	0	1	50.0	1	50.0	2	0.8
	1	37	68.5	17	31.5	54	22.0
	2	88	72.1	34	27.9	122	49.6
	3	45	66.2	23	33.8	68	27.6
	all	171	69.5	75	30.5	246	100.0
Tumor-adjacent	0	0	-	0	-	0	0.0
	1	36	97.3	1	2.7	37	15.0
	2	105	84.0	20	16.0	125	50.8
	3	49	58.3	35	41.7	84	34.1
	all	190	77.2	56	22.8	246	100.0

Table S4: Agreement of the Rep and CD68 staining intensity (INT) (A) and Rep and CD68 spread of the staining (POS) (B) by two independent raters.

Variable	Levels	n0	%0	n1	%1	n2	%2	nall	%all
age	37-55	0	0.0	10	12.1	15	9.3	25	10.2
	56-65	2	100.0	25	30.1	50	31.1	77	31.3
	66-79	0	0.0	38	45.8	69	42.9	107	43.5
	80-89	0	0.0	10	12.1	27	16.8	37	15.0
	all	2	100.0	83	100.0	161	100.0	246	100.0
sex	female	0	0.0	34	41.0	75	46.6	109	44.3
	male	2	100.0	49	59.0	86	53.4	137	55.7
	all	2	100.0	83	100.0	161	100.0	246	100.0
tumor localization	colon	1	50.0	41	49.4	110	68.3	152	61.8
	rectum	1	50.0	42	50.6	51	31.7	94	38.2
	all	2	100.0	83	100.0	161	100.0	246	100.0
tumor stage	1	0	0.0	21	25.3	25	15.5	46	18.8
	2	1	50.0	27	32.5	56	34.8	84	34.3
	3	1	50.0	25	30.1	56	34.8	82	33.5
	4	0	0.0	9	10.8	24	14.9	33	13.5
	missing	0	0.0	1	1.2	0	0.0	1	0.4
	all	2	100.0	83	100.0	161	100.0	246	100.0
MSI	no	2	100.0	75	90.4	140	87.0	217	89.7
	yes	0	0.0	5	6.0	20	12.4	25	10.3
	missing	0	0.0	3	3.6	1	0.6	4	1.6
	all	2	100.0	83	100.0	161	100.0	246	100.0
therapy	yes	1	50.0	34	41.0	78	48.5	113	45.9
	no	1	50.0	49	59.0	83	51.5	133	54.1
	all	2	100.0	83	100.0	161	100.0	246	100.0
milk	no	0	0.0	20	24.1	50	31.1	70	28.7
	low	1	50.0	25	30.1	38	23.6	64	26.2
	medium	1	50.0	16	19.3	29	18.0	46	18.9
	high	0	0.0	22	26.5	42	26.1	64	26.2
	missing	0	0.0	0	0.0	2	1.2	2	0.8
	all	2	100.0	83	100.0	161	100.0	246	100.0
meat	low	0	0.0	10	12.1	19	11.8	29	11.9
	medium	2	100.0	38	45.8	79	49.1	119	48.8
	high	0	0.0	35	42.2	61	37.9	96	39.3
	missing	0	0.0	0	0.0	2	1.2	2	0.8
	all	2	100.0	83	100.0	161	100.0	246	100.0

Table S5: Distributions of clinical parameters stratified by the Rep staining intensity (INT) in the tumor-adjacent mucosa. Variable “therapy” specifies chemo- and/or radiotherapy. Categorization for consumption of milk (variable “milk”): “no”- no milk consumption, “low” - once or less than once a week, “medium” - at least several times a week, “high” - at least once a day. Categorization for consumption of meat (variable “meat”): “low” - once or less than once a week, “medium” - at least several times a week, “high” - at least once a day. MSI – microsatellite instability.

Variable	Levels	n0	%0	n1	%1	n2	%2	n3	%3	nall	%all
age	37-55	0	0.0	8	15.4	8	7.6	9	10.3	25	10.2
	56-65	2	100.0	19	36.5	36	34.3	20	23.0	77	31.3
	66-79	0	0.0	20	38.5	48	45.7	39	44.8	107	43.5
	80-89	0	0.0	5	9.6	13	12.4	19	21.8	37	15.0
	all	2	100.0	52	100.0	105	100.0	87	100.0	246	100.0
sex	female	0	0.0	22	42.3	51	48.6	36	41.4	109	44.3
	male	2	100.0	30	57.7	54	51.4	51	58.6	137	55.7
	all	2	100.0	52	100.0	105	100.0	87	100.0	246	100.0
tumor localization	colon	1	50.0	28	53.9	74	70.5	49	56.3	152	61.8
	rectum	1	50.0	24	46.1	31	29.5	38	43.7	94	38.2
	all	2	100.0	52	100.0	105	100.0	87	100.0	246	100.0
tumor stage	1	0	0.0	12	23.1	20	19.1	14	16.1	46	18.7
	2	1	50.0	19	36.5	37	35.2	27	31.0	84	34.1
	3	1	50.0	15	28.9	32	30.5	34	39.1	82	33.3
	4	0	0.0	6	11.5	15	14.3	12	13.8	33	13.4
	missing	0	0.0	0	0.0	1	0.9	0	0.0	1	0.4
	all	2	100.0	52	100.0	105	100.0	87	100.0	246	100.0
MSI	no	2	100.0	49	94.2	91	86.7	75	86.2	217	88.2
	yes	0	0.0	3	5.8	12	11.4	10	11.5	25	10.2
	missing	0	0.0	0	0.0	2	1.9	2	2.3	4	1.6
	all	2	100.0	52	100.0	105	100.0	87	100.0	246	100.0
therapy	yes	1	50.0	21	40.0	49	46.7	42	48.3	113	45.9
	no	1	50.0	31	59.6	56	53.3	45	51.7	133	54.1
	all	2	100.0	52	100.0	105	100.0	87	100.0	246	100.0
milk	no	0	0.0	11	21.1	27	25.7	32	36.8	70	28.5
	low	1	50.0	14	26.9	30	28.6	19	21.8	64	26.0
	medium	1	50.0	15	28.9	15	14.3	15	17.2	46	18.7
	high	0	0.0	12	23.1	33	31.4	19	21.8	64	26.0
	missing	0	0.0	0	0.0	0	0.0	2	2.3	2	0.8
	all	2	100.0	52	100.0	105	100.0	87	100.0	246	100.0
meat	low	0	0.0	9	17.3.	11	10.5	9	10.3	29	11.8
	medium	2	100.0	22	42.3	54	51.4	41	47.1	119	48.8
	high	0	0.0	21	40.4	40	38.1	35	40.2	96	39.0
	missing	0	0.0	0	0.0	0	0.0	2	2.3	2	0.8
	all	2	100.0	52	100.0	105	100.0	87	100.0	246	100.0

Table S6: Distributions of clinical parameters stratified by the spread of the Rep staining (POS) in the tumor-adjacent mucosa. Variable “therapy” specifies chemo- and/or radiotherapy. Categorization for consumption of milk (variable “milk”): “no”- no milk consumption, “low” - once or less than once a week, “medium” - at least several times a week, “high” - at least once a day. Categorization for consumption of meat (variable “meat”): “low” - once or less than once a week, “medium” - at least several times a week, “high” - at least once a day. MSI – microsatellite instability.

Variable	Levels	n0	%0	n1	%1	n2	%2	nall	%all
age	37-55	14	11.0	9	9.6	2	8.0	25	10.2
	56-65	43	33.9	25	26.6	9	36.0	77	31.3
	66-79	50	39.4	47	50.0	10	40.0	107	43.5
	80-89	20	15.8	13	13.8	4	16.0	37	15.0
	all	127	100.0	94	100.0	25	100.0	246	100.0
sex	female	59	46.5	43	43.6	9	36.0	109	44.3
	male	68	53.5	53	56.4	16	64.0	137	55.7
	all	127	100.0	94	100.0	25	100.0	246	100.0
tumor localization	colon	82	64.6	54	57.5	16	64.0	152	61.8
	rectum	45	35.4	40	42.5	9	36.0	94	38.2
	all	127	100.0	94	100.0	25	100.0	246	100.0
tumor stage	1	20	15.8	22	23.4	4	16.0	46	18.7
	2	49	38.6	27	28.7	8	32.0	84	34.1
	3	36	28.4	35	37.2	11	44.0	82	33.3
	4	21	16.5	10	10.6	2	8.0	33	13.4
	missing	1	0.8	0	0.0	0	0.0	1	0.4
	all	127	100.0	94	100.0	25	100.0	246	100.0
MSI	no	110	86.6	85	90.4	22	88.0	217	88.2
	yes	15	11.8	8	8.5	2	8.0	25	10.2
	missing	2	1.6	1	1.1	1	4.0	4	1.6
	all	127	100.0	94	100.0	25	100.0	246	100.0
therapy	yes	54	42.5	50	53.2	9	36.0	113	45.9
	no	73	57.5	44	46.8	16	64.0	133	54.1
	all	127	100.0	94	100.0	25	100.0	246	100.0
milk	no	30	23.6	32	34.0	8	32.0	70	28.5
	low	35	27.6	21	22.3	8	32.0	64	26.0
	medium	27	21.3	16	17.0	3	12.0	46	18.7
	high	34	26.8	24	25.5	6	24.0	64	26.0
	missing	1	0.8	1	1.1	0	0.0	2	0.8
	all	127	100.0	94	100.0	25	100.0	246	100.0
meat	low	14	11.0	15	16.0	0	0.0	29	11.8
	medium	63	49.6	43	45.7	13	52.0	119	48.4
	high	49	38.6	35	37.2	12	48.0	96	39.0
	missing	1	0.8	1	1.1	0	0.0	2	0.8
	all	127	100.0	94	100.0	25	100.0	246	100.0

Table S7: Distributions of clinical parameters stratified by the Rep staining intensity (INT) in tumor tissues. Variable "therapy" specifies chemo- and/or radiotherapy. Categorization for consumption of milk (variable "milk"): "no"- no milk consumption, "low" - once or less than once a week, "medium" - at least several times a week, "high" - at least once a day. Categorization for consumption of meat (variable "meat"): "low" - once or less than once a week, "medium" - at least several times a week, "high" - at least once a day. MSI – microsatellite instability.

Variable	Levels	n0	%0	n1	%1	n2	%2	n3	%3	nall	%all
age	37-55	14	11.0	2	3.7	7	13.5	2	15.4	25	10.2
	56-65	43	33.9	18	33.3	14	26.9	2	15.4	77	31.3
	66-79	50	39.4	27	50.0	22	42.3	8	61.5	107	43.5
	80-89	20	15.8	7	13.0	9	17.3	1	7.7	37	15.0
	all	127	100.0	54	100.0	52	100.0	13	100.0	246	100.0
sex	female	59	46.5	23	42.6	23	44.2	4	30.8	109	44.3
	male	68	53.5	31	57.4	29	55.8	9	69.2	137	55.7
	all	127	100.0	54	100.0	52	100.0	13	100.0	246	100.0
tumor localization	colon	82	64.6	35	64.8	30	57.7	5	38.5	152	61.8
	rectum	45	35.4	19	35.2	22	42.3	8	61.5	94	38.2
	all	127	100.0	54	100.0	52	100.0	13	100.0	246	100.0
tumor stage	1	20	15.8	11	20.4	10	19.2	5	38.5	46	18.7
	2	49	38.6	16	29.6	15	28.9	4	30.8	84	34.1
	3	36	28.4	22	40.7	20	38.5	4	30.8	82	33.3
	4	21	16.5	5	9.3	7	13.5	0	0.0	33	13.4
	missing	1	0.8	0	0.0	0	0.0	0	0.0	1	0.4
	all	127	100.0	54	100.0	52	100.0	13	100.0	246	100.0
MSI	no	110	86.6	48	88.9	48	92.3	11	84.6	217	88.2
	yes	15	11.8	5	9.3	3	5.8	2	15.4	25	10.2
	missing	2	1.6	1	1.9	1	1.9	0	0.0	4	1.6
	all	127	100.0	54	100.0	52	100.0	13	100.0	246	100.0
therapy	yes	54	42.5	29	53.7	25	48.1	5	38.5	113	45.9
	no	73	57.5	25	46.3	27	51.9	8	61.5	133	54.1
	all	127	100.0	54	100.0	52	100.0	13	100.0	246	100.0
milk	no	30	23.6	17	31.5	20	38.5	3	23.1	70	28.5
	low	35	27.6	12	22.2	13	25.0	4	30.8	64	26.0
	medium	27	21.3	8	14.8	8	15.4	3	23.1	46	18.7
	high	34	26.8	16	29.6	11	21.1	3	23.1	64	26.0
	missing	1	0.8	1	1.9	0	0.0	0	0.0	2	0.8
	all	127	100.0	54	100.0	52	100.0	13	100.0	246	100.0
meat	low	14	11.0	11	20.4	4	7.7	0	0.0	29	11.8
	medium	63	49.6	19	35.2	29	55.8	8	61.5	119	48.4
	high	49	38.6	23	42.6	19	36.5	5	38.5	96	39.0
	missing	1	0.8	1	1.9	0	0.0	0	0.0	2	0.8
	all	127	100.0	54	100.0	52	100.0	13	100.0	246	100.0

Table S8: Distributions of clinical parameters stratified by the spread of the Rep staining (POS) in tumor tissue. Variable “therapy” specifies chemo- and/or radiotherapy. Categorization for consumption of milk (variable “milk”): “no”- no milk consumption, “low” - once or less than once a week, “medium” - at least several times a week, “high” - at least once a day. Categorization for consumption of meat (variable “meat”): “low” - once or less than once a week, “medium” - at least several times a week, “high” - at least once a day. MSI – microsatellite instability.

Variable	No.	Rep			
		Tumor		Tumor-adjacent	
		Rep INT	Rep POS	Rep INT	Rep POS
		P (*P* unadj.)	*P* (*P* unadj.)	*P* (*P* unadj.)	*P* (*P* unadj.)
Age					
37-55	25	1.000 (0.571)	1.000 (0.585)	1.000 (0.438)	0.111 **(0.014)**
56-65	77				
66-79	107				
80-89	37				
Sex					
Female	109	1.000 (0.393)	1.000 (0.432)	1.000 (0.290)	1.000 (0.893)
Male	137				
Localization					
Colon	152	1.000 (0.463)	1.000 (0.165)	**0.031 (0.004)**	1.000 (0.832)
Rectum	94				
Stage					
1	46	1.000 (0.691)	1.000 (0.461)	0.818 (0.117)	1.000 (0.204)
2	84				
3	82				
4	33				
MSI					
No	217	1.000 (0.390)	1.000 (0.414)	0.818 (0.119)	1.000 (0.301)
Yes	25				
Therapy					
Yes	113	1.000 (0.507)	1.000 (0.470)	1.000 (0.286)	1.000 (0.425)
No	133				
Milk					
No	70	0.242 (1.000)	1.000 (0.199)	1.000 (0.606)	0.818 (0.117)
Low	64				
Medium	46				
High	64				
Meat					
Low	29	1.000 (0.719)	1.000 (0.909)	1.000 (0.795)	1.000 (0.545)
Medium	119				
High	96				

Table S9: Association of Rep staining intensity (INT) and spread on the staining (POS) in tumor and tumor-adjacent mucosa of CRC patients with clinicopathological parameters (age, sex, tumor localization and stage, MSI state and therapy at diagnosis, including milk and meat consumption). *P*-values with Holm correction for multiple testing or without ($P_{unadjusted}$) for association of ordinal (age, stage, milk, meat) and nominal clinical parameters (gender, localization, therapy, microsatellite instability - MSI) with Rep INT or POS. Test for ordinal parameters versus INT/POS: Jonckheere-Terpstra test; test for nominal parameters: Kruskall-Wallis test.

Variable	No.	CD68			
		Tumor		Tumor-adjacent	
		CD68 INT	CD68 POS	CD68 INT	CD68 POS
		P (*P* unadj.)	*P* (*P* unadj.)	*P* (*P* unadj.)	*P* (*P* unadj.)
Age					
37-55	25	1.000 (0.761)	1.000 (0.573)	0.642 (0.092)	1.000 (0.238)
56-65	77				
66-79	107				
80-89	37				
Sex					
Female	109	0.911 (0.114)	1.000 (0.255)	1.000 (0.811)	1.000 (0.391)
Male	137				
Localization					
Colon	152	1.000 (0.246)	0.647 (0.081)	1.000 (0.592)	1.000 (0.360)
Rectum	94				
Stage					
1	46	1.000 (0.518)	1.000 (0.427)	1.000 (0.256)	1.000 (0.266)
2	84				
3	82				
4	33				
MSI					
No	217	1.000 (0.900)	1.000 (0.832)	1.000 (0.231)	1.000 (0.199)
Yes	25				
Therapy					
Yes	113	1.000 (0.451)	1.000 (0.793)	0.405 (0.051)	1.000 (0.356)
No	133				
Milk					
No	70	1.000 (0.914)	1.000 (0.208)	1.000 (0.862)	0.841 (0.105)
Low	64				
Medium	46				
High	64				
Meat					
Low	29	1.000 (0.817)	1.000 (0.610)	1.000 (0.504)	1.000 (0.577)
Medium	119				
High	96				

Table S10: Association of CD68 staining intensity (INT) and spread of the staining (POS) in tumor and tumor-adjacent mucosa of CRC patients with clinicopathological parameters (age, sex, tumor localization and stage, MSI state and therapy at diagnosis, including milk and meat consumption). *P*-values with Holm correction for multiple testing or without ($P_{unadjusted}$) for association of ordinal (age, stage, milk, meat) and nominal clinical parameters (gender, localization, therapy, microsatellite instability - MSI) with Rep INT or POS. Test for nominal and ordinal parameters versus INT/POS: Kruskall-Wallis test and Jonckheere-Terpstra test, respectively.

A Variable	Levels	n0	%0	ncrc	%crc	nother	%other	nall	%all
Rep INT	0	58	52.2	41	53.2	26	47.3	127	51.6
tumor	1	41	36.9	28	36.4	24	43.6	94	38.2
	2	12	10.8	8	10.4	5	9.1	25	10.2
	all	111	100.0	77	100.0	55	100.0	246	100.0
CD68 INT	0	1	0.9	1	1.3	0	0.0	2	0.8
tumor	1	46	41.4	36	46.8	23	41.8	107	43.5
	2	64	57.7	40	52.0	32	58.2	137	55.7
	all	111	100.0	77	100.0	55	100.0	246	100.0
Rep INT	0	1	0.9	0	0.0	1	1.8	2	0.8
tum.-adj.	1	36	32.4	26	33.8	21	38.2	83	33.7
mucosa	2	74	66.7	51	66.2	33	60.0	161	65.5
	all	111	100.0	77	100.0	55	100.0	246	100.0
CD68 INT	0	0	-	0	-	0	-	0	0.0
tum.-adj.	1	13	11.7	7	9.1	7	12.7	28	11.4
mucosa	2	98	88.3	70	90.9	48	87.3	218	88.6
	all	111	100.0	77	100.0	55	100.0	246	100.0

B Variable	Levels	n0	%0	ncrc	%crc	nother	%other	nall	%all
Rep POS	0	58	52.2	41	53.2	26	47.3	127	51.6
tumor	1	21	18.9	17	22.1	16	29.1	54	21.9
	2	26	23.4	18	23.4	7	12.7	52	21.1
	3	6	5.4	1	1.3	6	10.9	13	5.3
	all	111	100.0	77	100.0	55	100.0	246	100.0
CD68 POS	0	1	0.9	1	1.3	0	0.0	2	0.8
tumor	1	21	18.9	23	29.9	9	16.4	54	21.9
	2	55	49.5	37	48.0	30	54.5	122	49.6
	3	34	30.6	16	20.8	16	29.1	68	27.6
	all	111	100.0	77	100.0	55	100.0	246	100.0
Rep POS	0	1	0.9	0	0.0	1	1.8	2	0.8
tum.-adj.	1	23	20.7	12	15.6	15	27.3	52	21.1
mucosa	2	55	49.5	32	41.6	17	30.9	105	42.7
	3	32	28.8	33	42.9	22	40.0	87	35.4
	all	111	100.0	77	100.0	55	100.0	246	100.0
CD68 POS	0	0	-	0	-	0	-	0	0.0
tum.-adj.	1	18	16.2	9	11.7	9	16.4	37	15.0
mucosa	2	61	55.0	34	44.2	29	52.7	125	50.8
	3	32	28.8	34	44.2	17	30.9	84	34.1
	all	111	100.0	77	100.0	55	100.0	246	100.0

Table S11: Distribution of Rep and CD68 staining intensity (INT) (A) and spread of the staining (POS) (B) stratified by censored (n0), CRC-specific death (n_{CRC}), non-CRC-specific death (n_{other}) and unstratified (n_{all}). tum.-adj. – tumor-adjacent.

	CRC-specific death					non-CRC-specific death				
	HR	CI (lower)	CI (upper)	P		HR	CI (lower)	CI (upper)	P	
age	1.00	0.97	1.03	0.989		1.10	1.06	1.14	<0.001	***
sex: male	0.52	0.3	0.89	0.017	*	1.02	0.52	1.98	0.954	
tumor localization: rectal	1.44	0.83	2.51	0.198		0.87	0.42	1.83	0.721	
tumor stage: 2	2.79	0.77	10.14	0.12		0.82	0.35	1.92	0.640	
tumor stage: 3	9.64	2.39	38.91	0.001	**	0.96	0.31	3.01	0.941	
tumor stage: 4	35.41	8.91	140.63	<0.001	***	0.46	0.05	4.22	0.491	
MSI: yes	0.64	0.21	1.88	0.413		1.46	0.57	3.73	0.433	
therapy: no	1.45	0.68	3.08	0.332		1.20	0.47	3.10	0.705	
milk: low	0.79	0.41	1.51	0.475		0.81	0.36	1.82	0.604	
milk: medium	0.60	0.29	1.28	0.193		1.00	0.40	2.54	0.996	
milk: high	0.54	0.28	1.06	0.075	.	0.71	0.31	1.65	0.430	
meat: medium	1.43	0.62	3.29	0.397		0.84	0.31	2.27	0.733	
meat: high	1.33	0.55	3.20	0.529		0.81	0.28	2.31	0.696	
tumor CD68 POS: 2	0.64	0.34	1.22	0.175		1.49	0.61	3.64	0.382	
tumor CD68 POS: 3	0.58	0.27	1.24	0.157		1.30	0.49	3.40	0.600	
tumor CD68 INT: 2	1.22	0.72	2.08	0.460		1.06	0.58	1.95	0.841	
tum.-adj. CD68 POS: 2	1.19	0.50	2.84	0.690		0.82	0.30	2.24	0.695	
tum.-adj. CD68 POS: 3	1.71	0.62	4.72	0.303		0.81	0.24	2.78	0.743	
tum.-adj. CD68 INT: 2	1.21	0.45	3.22	0.707		0.59	0.19	1.80	0.355	
tumor Rep POS: 2	0.93	0.47	1.85	0.840		0.60	0.24	1.52	0.280	
tumor Rep POS: 3	0.27	0.03	2.30	0.232		1.24	0.39	3.91	0.719	
tumor Rep INT: 2	1.14	0.46	2.81	0.780		1.38	0.41	4.65	0.600	
tum.-adj. Rep POS: 2	1.12	0.49	2.53	0.789		0.36	0.15	0.85	0.020	*
tum.-adj. Rep POS: 3	1.37	0.54	3.49	0.504		1.01	0.35	2.97	0.980	
tum.-adj. Rep INT: 2	0.85	0.49	1.48	0.566		0.87	0.44	1.72	0.658	

Table S12: Cause-specific proportional hazard models combined for Rep and CD68 for CRC-specific and non-CRC-specific death. Reference category for sex: “male”: sex: “female”, reference category for tumor localization: “rectum”: tumor localization: “colon”, reference category for therapy: “yes”: therapy: “no”, reference category for tumor stage: “2” “3” “4”: tumor stage: “1”, reference category for MSI: “yes”: MSI: “no”, reference category for milk: “low” “medium” “high”: milk: ”no”, reference category for meat: “medium” “high”: meat: “low”, reference category for tumor or tumor-adjacent Rep and CD68 staining intensity (INT) or spread of the staining (POS) “2” “3”: “0/1”. MSI - microsatellite instability, tum.-adj. – tumor-adjacent.

References

1. Bund T, Nikitina E, Chakraborty D, Ernst C, Gunst K, Boneva B, Tessmer C, Volk N, Brobeil A, Weber A, Heikenwalder M, zur Hausen H, de Villiers EM. Analysis of chronic inflammatory lesions of the colon for BMMF Rep antigen expression and CD68 macrophage interactions. *Proc Natl Acad Sci USA* 2021;**118**(12):e2025830118.
